# Occurrence of Bacterial Pathogens and Human Noroviruses in Shellfish-Harvesting Areas and Their Catchments in France

**DOI:** 10.3389/fmicb.2018.02443

**Published:** 2018-10-11

**Authors:** Alain Rincé, Charlotte Balière, Dominique Hervio-Heath, Joëlle Cozien, Solen Lozach, Sylvain Parnaudeau, Françoise S. Le Guyader, Simon Le Hello, Jean-Christophe Giard, Nicolas Sauvageot, Abdellah Benachour, Sofia Strubbia, Michèle Gourmelon

**Affiliations:** ^1^UNICAEN, U2RM, Normandie Université, Caen, France; ^2^RBE-SG2M-LSEM, Institut Français de Recherche pour l’Exploitation de la Mer, Brest, France; ^3^Unité des Bactéries Pathogènes Entériques, Institut Pasteur,Paris, France

**Keywords:** *Campylobacter*, *Salmonella*, *Vibrio*, HuNoVs, fecal bacterial indicators, shellfish, water

## Abstract

During a 2-year study, the presence of human pathogenic bacteria and noroviruses was investigated in shellfish, seawater and/or surface sediments collected from three French coastal shellfish-harvesting areas as well as in freshwaters from the corresponding upstream catchments. Bacteria isolated from these samples were further analyzed. *Escherichia coli* isolates classified into the phylogenetic groups B2, or D and enterococci from *Enterococcus faecalis* and *E. faecium* species were tested for the presence of virulence genes and for antimicrobial susceptibility. *Salmonella* members were serotyped and the most abundant serovars (Typhimurium and its monophasic variants and Mbandaka) were genetically characterized by high discriminative subtyping methods. *Campylobacter* and *Vibrio* were identified at the species level, and haemolysin-producing *Vibrio parahaemolyticus* were searched by *tdh*- and *trh*- gene detection. Main results showed a low prevalence of *Salmonella* in shellfish samples where only members of *S.* Mbandaka were found. *Campylobacter* were more frequently isolated than *Salmonella* and a different distribution of *Campylobacter* species was observed in shellfish compared to rivers, strongly suggesting possible additional inputs of bacteria. Statistical associations between enteric bacteria, human noroviruses (HuNoVs) and concentration of fecal indicator bacteria revealed that the presence of *Salmonella* was correlated with that of *Campylobacter jejuni* and/or *C. coli* as well as to *E. coli* concentration. A positive correlation was also found between the presence of *C. lari* and the detection of HuNoVs. This study highlights the importance of simultaneous detection and characterization of enteric and marine pathogenic bacteria and human noroviruses not only in shellfish but also in catchment waters for a hazard assessment associated with microbial contamination of shellfish.

## Introduction

Shellfish and coastal waters contaminated by human pathogens could be sources of shellfish-borne or water-borne outbreaks (Potasman et al., 2002; Yoder et al., 2008). In fact, shellfish can accumulate and concentrate microbial pathogens present in waters by their filter-feeding activities. Because shellfish are often consumed raw or lightly cooked, they present a health risk for exposure to bacterial and viral pathogens. Indeed, crustaceans, shellfish, mollusks and products thereof constitute food vehicles in 7.3% of the strong-evidence outbreaks in the European Union (EU) in 2013 (European Food Safety Authority [EFSA] and European Centre for Disease Prevention Control [ECDC], 2015). The microbiological quality of shellfish-harvesting areas and bathing waters is assessed by enumeration of the fecal indicator bacteria (FIBs) *Escherichia coli* and enterococci. Their presence can lead to closures or downgrading of shellfish-harvesting areas and bathing areas. These FIBs inform of the possible presence of fecal microorganisms potentially pathogenic for humans such as members of *E. coli* species or *Enterococcus* genus, zoonotic bacteria such as *Salmonella* spp. and *Campylobacter* spp. which could originate from urban and agricultural catchments upstream coastal bathing waters and shellfish-harvesting areas. However, these indicators do not take into account the risk associated with the presence of marine bacteria such as *Vibrio* or HuNoVs which are also responsible for outbreaks from shellfish origin (DePaola et al., 2010; Amagliani et al., 2012; Yu et al., 2015).

Numerous bacterial human pathogens are present in coastal waters and shellfish (Escobedo-Hinojosa and Pardo-López, 2017; Leight et al., 2018). They could be classified as allochthonous bacteria, coming from upstream catchments or as autochthonous such as *Vibrio*. Among allochthonous bacteria, some are of fecal origin such as members of Enterobacteriaceae (e.g., pathogenic *E. coli*, *Salmonella*), pathogenic enterococci, *Campylobacter* and others are from aquatic environment and soil such as *Aeromonas, Arcobacter*, and *Pseudomonas.*

A selection of these pathogens was investigated in this study, starting with pathogenic bacteria among the FIB. In fact, even commensals of the intestinal microflora of humans and animals, some strains of *E. coli* such that producing Shiga-toxin (STEC) or enteropathogenic *E. coli* (EPEC) are able to cause pathologies that could potentially lead to hemolytic uremic syndrome. Members of *E. coli* are also responsible for extra-intestinal pathologies including urinary tract infections, meningitis or septicemia (Kaper et al., 2004). *E. coli* can easily be divided into four main phylogenetic groups (A, B1, B2, and D) (Clermont et al., 2000). Extraintestinal pathogenic *E. coli* (ExPEC) mainly belong to groups B2 and D whereas most commensal strains belong to groups A and B1 (Picard et al., 1999; Johnson et al., 2001). Since the last decades, enterococci have become nosocomial pathogens of global importance where *Enterococcus faecium* and *E. faecalis* are clinically the most feared species (Guzman Prieto et al., 2016). Moreover, *E. coli* and enterococci may contribute to the dissemination of antibiotic resistance genes (Boehm and Sassoubre, 2014; Andrade et al., 2015).

Among fecal pathogens, we also focus on bacteria mainly coming from livestock animals upstream coastal areas such as non-typhoidal *Salmonella* and *Campylobacter* spp. These bacteria are leading causes of bacterial gastroenteritis in many countries. With norovirus, they accounted for more than 70% of food-borne pathogens-associated illnesses and hospitalizations in France (Van Cauteren et al., 2017). Salmonellosis can be due to numerous serovars of *Salmonella enterica* subspecies *enterica* but few of them (such as Typhimurium and its monophasic variants or Enteritidis) are cause of most human infections (European Food Safety Authority [EFSA] and European Centre for Disease Prevention Control [ECDC], 2016). *Salmonella* spp. are present in poultry, cattle, pigs, and wild birds and also isolated from fresh and marine waters (Gorski et al., 2011; Walters et al., 2013; Flockhart et al., 2017). While *S.* Typhimurium is ubiquitous, *S.* Dublin and *S.* Enteritidis are more commonly found in cattle and in poultry, respectively (David et al., 2013; European Food Safety Authority [EFSA] and European Centre for Disease Prevention Control [ECDC], 2016). With over 229,000 human cases a year, campylobacteriosis is the most frequently reported food-borne illness in EU mainly due to *Campylobacter jejuni* followed, by far by *C. coli*, whereas *C. lari* is implicated in a lesser extend (European Food Safety Authority [EFSA] and European Centre for Disease Prevention Control [ECDC], 2016). Animals are the main reservoirs of *Campylobacter*. More specifically, *C. jejuni*, *C. coli*, and *C. lari* are mainly found in poultry and cattle, in poultry and swine, and in gulls and shorebirds, respectively (Denis et al., 2011a; Miller et al., 2014; Pitkanen and Hanninen, 2017). *Campylobacter* has been frequently detected in waters at the level of catchments and in coastal areas (Martinez-Urtaza et al., 2004; Van Dyke et al., 2010; Wilkes et al., 2011). Few shellfish outbreaks due to *Campylobacter* have been reported (Abeyta et al., 1993).

Among the allochthonous bacterial pathogens, *Vibrio* spp. occurring naturally in brackish and marine waters, *Vibrio parahaemolyticus*, *V. vulnificus*, and *V. cholerae* were selected for this study as they are the main species involved in seafood- and seawater-borne illness worldwide^1^. These *Vibrio* species are commonly isolated in coastal waters from France and Europe and the risk of further *Vibrio* infections might increase in the coming decades due to climate change (Hervio-Heath et al., 2002; Martinez-Urtaza et al., 2012; Esteves et al., 2015; Semenza et al., 2017).

Finally, in addition to human bacterial pathogens, enteric viruses such as HuNoVs, were also selected as they are the main cause of gastroenteritis worldwide. HuNoVs are a group of highly diverse viruses that belong to the *Caliciviridae* family. They are non-enveloped icosahedral viruses with a single stranded RNA genome. HuNoVs cause gastroenteritis characterized by vomiting, abdominal cramps, fever, watery diarrhea, headaches, chills and myalgia, and illness normally lasts 2–3 days. They are currently classified in seven genogroups, of which three infect humans (GI, GII, and GIV) (de Graaf et al., 2016). HuNoV is one of the most infectious pathogens, as demonstrated through human volunteer studies, or analysis of data from shellfish-related outbreaks (Thebault et al., 2013; Atmar et al., 2014). GI and GII HuNoVs are excreted at very high levels in the feces (up to 10^11^ copies/g) of both symptomatic and asymptomatic people during long periods (at least 27 days) (Atmar et al., 2014; Miura et al., 2017).

The aim of the present study was to detect and characterize microorganisms from shellfish-harvesting areas and their catchments in order to assess the prevalence and diversity of selected pathogenic bacteria (*Salmonella*, *Campylobacter*, and *Vibrio*), FIBs and HuNoVs. For this purpose, samples of shellfish batches, freshwater, seawater and surface sediments collected monthly over a 2-year period in three selected shellfish-harvesting areas and their catchments were analyzed. This study presents the interest of focusing on the simultaneous detection and/or isolation of pathogenic enteric and marine bacteria, and HuNoVs in coastal environments (shellfish and waters from the catchment).

## Materials and Methods

### Sampling Locations and Sample Description

**FIGURE 1:**
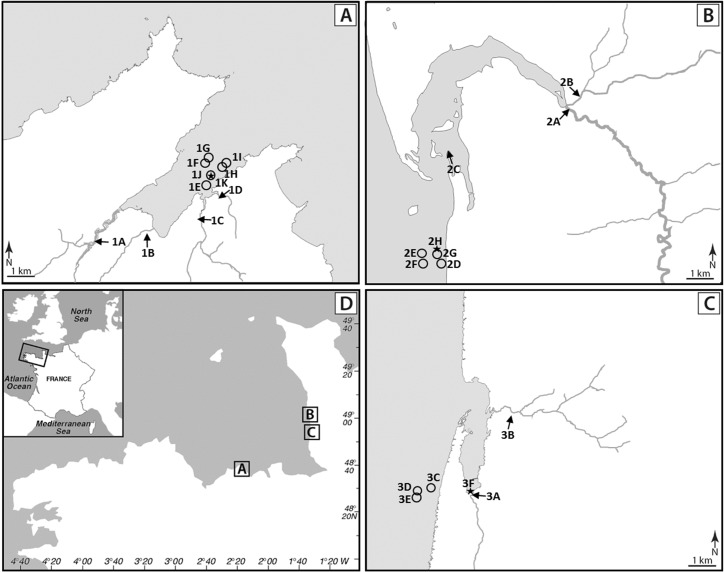
Location of sampling sites. **(A)** La Fresnaye (Brittany) site 1. **(B)** Regnéville sur mer (Normandy) site 2. **(C)** La Vanlée (Normandy) site 3. **(D)** Location of studied sites on a map of France. Sampling sites are represented by arrows (water from river and brackish water), circles (shellfish and seawater) and stars (sediment).

A total of 505 samples including 237 shellfish batches, 40 surface sediments, 12 seawaters and 216 waters from nine sampling points upstream of shellfish-harvesting areas described in Balière et al. (2015) were analyzed. These samples came from three shellfish-harvesting sites located on the English Channel coast and in their catchments. On the catchment of the Brittany site (site 1, La Fresnaye), four rivers flow with their outlet in the bay: Frémur (1A, 50 km long, sub-catchment: 77.4 km^2^), Le Rat (1B, 10.5 km, sub-catchment: 19.2 km^2^), Le Clos (1C, 7 km, sub-catchment: 13.3 km^2^) and Kermiton (1D, 2.3 km, sub-catchment: 6.3 km^2^) (**Figure 1**). Cattle, swine and poultry are intensively bred on this catchment (**Supplementary Data S1**). The Normandy site 2 (Regnéville sur mer) is characterized by the intensive breeding of cattle and sheep and by the presence of poultry and swine, while its neighboring site 3 (La Vanlée) rather hosts cattle, sheep, and swine (**Supplementary Data S1**). The main rivers on these sites are La Sienne (2A, 93 km, sub-catchment: 794 km^2^) and La Soules (2B, 53 km, sub-catchment: 150 km^2^) on site 2, and La Vanlée (3A, 8.5 km, sub-catchment: 35 km^2^) and Les Hardes (3B, 6 km, sub-catchment: 15 km^2^) on site 3.

### Preparation of Samples

Shellfish [oyster, mussel, and common cockle batches (site 1, *n* = 120; site 2, *n* = 71 and site 3, *n* = 46)] and waters from sampling points upstream of shellfish-harvesting areas (site 1, *n* = 96; site 2, *n* = 72; site 3, *n* = 48) were collected monthly from February 2013 to January 2015, whereas surface sediment samples (site 1, *n* = 14; site 2, *n* = 13; site 3, *n* = 13) were collected from February 2013 to January 2014 and seawater samples (site 1, *n* = 12) from February 2014 to January 2015 (Balière et al., 2015). If all the samples taken from the rivers Frémur, Le Rat, Le Clos, La Soules, and Les Hardes had a salinity of less than 1 g/L, one from the mouth of the river La Sienne and several samples from the estuary of Regnéville sur mer and Kermiton presented a higher salinity.

Water samples were analyzed by membrane filtration and shellfish were opened and dissected aseptically for analyses. After opening, 25 g of homogenized blended total shellfish flesh (SF; including shellfish flesh and intravalvular liquid) were introduced into 225 ml of the appropriate enrichment buffer according to the targeted bacteria. For sediment, 10 g were introduced into the appropriate buffer, while for water samples 1 L was filtered using the appropriate cellulose membranes (0.45 μm for *Salmonella* and *E. coli* and 0.2 μm for *Campylobacter* and *Vibrio*) and the filters were placed in the appropriate enrichment buffer.

### Detection and Characterization of *E. coli* and Enterococci

*Escherichia coli* and enterococci from water samples were enumerated by ISO-9308-3:1998 (1998) and ISO-7899-1:1998 (1998) methods, respectively, and *E. coli* from shellfish batches by impedance method according to AFNOR-NF-V-08-106:2010 (2010). In addition, *E. coli* and enterococci were isolated as described in **Supplementary Data S2**. DNAs were extracted using Instagene matrix (Bio-Rad) after mechanical lysis of the bacteria with glass micro-beads.

*Escherichia coli* isolates were classified into the four main phylogenetic groups A, B1, B2, or D using a method based on the detection of two genes, *chuA* and *yjaA* and of a DNA fragment designated TSPE4.C2 (Clermont et al., 2000). Detection of these elements was performed by quantitative PCR (qPCR) using SYBER Green precision melt supermix (Bio-Rad) and primers described in **Supplementary Data S3**. Detection of *stx* and *eae* genes, encoding Shiga-toxin and intimin, respectively, and isolation of strains belonging to the STEC and EPEC pathotypes which are frequently observed as being responsible for diarrhea were the subject of a previous study (Balière et al., 2015). Thus, we focused here to detection of virulence genes characteristic of ExPEC. The presence of genes which encode fimbriae (*papC* and *sfa*), adhesins (*papGII* and *papGIII*), hemolysin (*hlyC*), cytotoxic necrotizing factor (*cnf1*), siderophore synthesis (*iucC*) and siderophore receptors (*fyuA* and *iroN*) was investigated by PCR using primers and protocol previously described by Bonacorsi et al. (2006) and GoTaq Flexi DNA polymerase (Promega).

Presence of virulence genes in enterococci [i.e., *esp*, *gelE*, and *agg* encoding the extracellular protein Esp (Shankar et al., 1999), an extracellular zinc metallo-endopeptidase (Su et al., 1991), and an aggregation substance (Kreft et al., 1992), respectively] was determined by multiplex qPCR using SYBER Green precision melt supermix and primers described in **Supplementary Data S3**.

Antimicrobial susceptibility testing of a selection of 556 *E. coli* and 446 enterococci (213 *E. faecalis* and 233 *E. faecium*) isolates was based on the disk diffusion method on Muller-Hinton medium (AES-chemunex, Bruz, France). Plates were incubated at 37°C for 24 h.

### Detection, Isolation and Characterization of *Salmonella*

*Salmonella* spp. were detected and isolated as described in the **Supplementary Data S2**.

Serotyping of *Salmonella* strains was performed by agglutination tests with antisera (Bio-Rad, Marnes-la-Coquette, France) according to the White–Kauffmann–Le Minor scheme (Grimont and Weill, 2007).

Depending on the serotypes, different subtyping methods were performed in order to get suitable and discriminative information on the main *Salmonella* populations that were circulating. For all *Salmonella* Typhimurium isolates (as well as their monophasic or non-motile variants), the CRISPR (Clustered Regularly Interspaced Short Palindromic Repeats) spacer content was recognized by a high-throughput method (named CRISPOL), as previously described (Fabre et al., 2012). DNA macrorestriction, i.e., Pulsenet standard *Xba*I-PFGE protocol (Ribot et al., 2006), and/or whole genome sequencing (WGS) were performed for *Salmonella* Mbandaka isolates. Sequencing and phylogenetic analyzes were done as previously described by Fonteneau et al. (2017). Furthermore, various genetic analyses, like Multi-Locus Sequence Typing (MLST) and acquired antibiotic resistance gene content determination, were determined on assembled sequences with web-based tools^2^ and by an in-house Perl script. For each isolate, the paired-end reads were aligned against the *S. enterica* serotype Mbandaka str. 9367/03 genome^3^.

### Detection, Isolation and Characterization of *Campylobacter*

*Campylobacter* spp. were detected and isolated as described in **Supplementary Data S2**.

Genomic DNA was extracted from colonies suspended in water using Nuclisens protocol (BioMérieux). Isolates were confirmed as *Campylobacter* spp. by detecting 16S rRNA gene by qPCR (Leblanc-Maridor et al., 2011). Species were identified by mass spectrometry using a MALDI-TOF Bruker Microflex apparatus (MALDI-TOF Bruker Microflex, Billerica, MA, United States) and following the protocol described by the supplier.

### Detection, Isolation and Characterization of *Vibrio*

Detection and isolation of *V. parahaemolyticus*, *V. cholerae*, and *V. vulnificus* in a selection of samples including shellfish and seawater were performed as described in **Supplementary Data S2**.

DNA was heat-released from APW-enriched cultures and presence of total and haemolysin-producing *V. parahaemolyticus* and of *V. cholerae* and *V. vulnificus* was detected by qPCR and PCR. Presumptive *V. parahaemolyticus*, *V. vulnificus* and *V. cholerae* colonies were selected from PCR-positive APW-enriched cultures streaked (previously) onto chromogenic (CHROMagar^TM^ Vibrio, Humeau) and selective (Thiosulfate Citrate Bile Salts Sucrose, TCBS, Difco) media plates and incubated 24 h at 37°C. These colonies were analyzed by qPCR and PCR using the primer sets described in **Supplementary Data S2**.

### Detection of Human Noroviruses

For detection of HuNoVs, shellfish were shucked, and the digestive tissues (DT) were recovered and frozen under aliquots of 2 g. For analysis, Mengovirus (MgV) (2.10^6^ RNA copies) was added to each sample and incubated with 2 ml of proteinase K solution (30 U/mg, Sigma-Aldrich, France) at 37°C under shaking for 15 min and then at 60°C for 15 min. After centrifugation at 3,000 *g* for 5 min, the supernatant was recovered. Nucleic acids (NAs) were extracted using the NucliSens extraction kit (BioMérieux) with increasing the lysis buffer volume (Le Mennec et al., 2017). After checking the extraction efficiency by amplification of MgV, HuNoVs GI and GII were detected as previously described using 5 μl of undiluted and 10-fold dilutions of the NA extract on a Mx3000P qPCR System (Agilent Technologies, France). Only sample with a MgV extraction efficiency >1% were considered for quantification. The number of RNA copies present in each positive evaluable sample was estimated by comparing the sample *C*q value to standard curves. Calculated concentrations for GI and GII were added to express the final result as HuNoV RNAc/g of DT.

### Environmental Data and Statistical Analysis

Rainfall data (2-days cumulative rainfall before sampling date) were provided by the meteorological stations from Meteo France at Pleurtuit (site 1) and at Coutances (sites 2 and 3). On-site water temperature, and salinity were measured using a multi sensor probe 3430 (WTW) equipped with a FDO 925 electrode and a pH-electrode Sentix 940 (site 1) or an EcoSence EC300 apparatus (VWR) (sites 2 and 3). Data on temperature and precipitation were categorized into three groups whose boundaries were defined to have a number of samples compatible with a reliable statistical analysis in each category. Comparisons of prevalence and microorganism’s characteristics between samples type, site, season, temperature and precipitation were analyzed by the chi-square test using the CHISQ.TEST function of the Excel software (Microsoft). A *P*-value of <0.05 was considered statistically significant. In case sample was positive for multiple pathogens of interest, it was considered positive for each of the pathogens in question.

The Shannon diversity index $\left[H^{\prime }=-\sum_{i=1}^{S}Pi\log_{2}(Pi)\right]$ was calculated using the Biodiversity Calculator tool^4^.

Odds ratios (OR) and relative risk (RR) were calculated using MEDCALC^5^ and considered statistically significant when *P*-values were less <0.05. OR is the odds of a positive sample for a given parameter among positive samples for a second parameter while RR is the probability that a sample is positive for a given parameter among samples positive for a second parameter relative to the probability that it is positive for the given parameter among samples negative for the second parameter.

## Results

### Enumeration of *E. coli* and Enterococci

**FIGURE 2:**
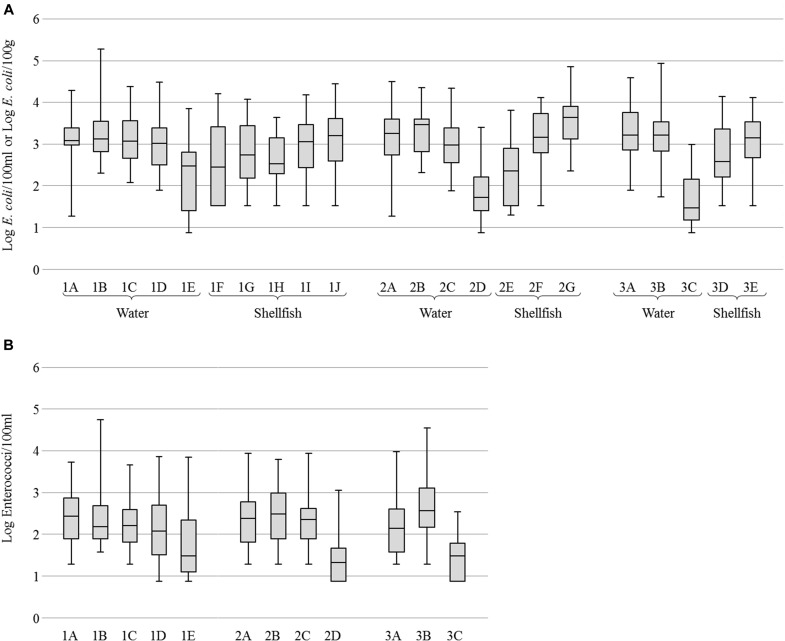
Enumeration of fecal indicator bacteria. **(A)** Boxplots of *Escherichia coli* concentration in water and shellfish. **(B)** Boxplots of Enterococci concentration in water. Boxplots show the minimum, the 25th percentile, the median, the 75th percentile and the maximum concentration. For water samples, 1E, 2D, and 3C correspond to seawater and others to river or brackish water. For shellfish samples, 1F, 1H, 2E, and 3D correspond to oysters, 1G, 1I, 2F, and 3E to mussels, and 1J and 2G to cockles.

For all sites, concentrations of *E. coli* were variable and generally high in river water (geometric mean (GM) of 3.2 Log per 100 ml) and cockle samples (GM of 3.3 Log per 100 g) while oysters, mussels and seawaters were less contaminated (GM of 2.6, 2.9 and 1.9 Log, respectively). Mussels were found more contaminated than oysters (**Figure 2A**). Concentrations of enterococci in water were also variable but lower than those of *E. coli* with GM from 2.1 to 3.6 Log of enterococci per 100 ml in river waters and from 1.4 to 1.7 Log in seawaters (**Figure 2B**).

### Characterization of *E. coli*

**FIGURE 3:**
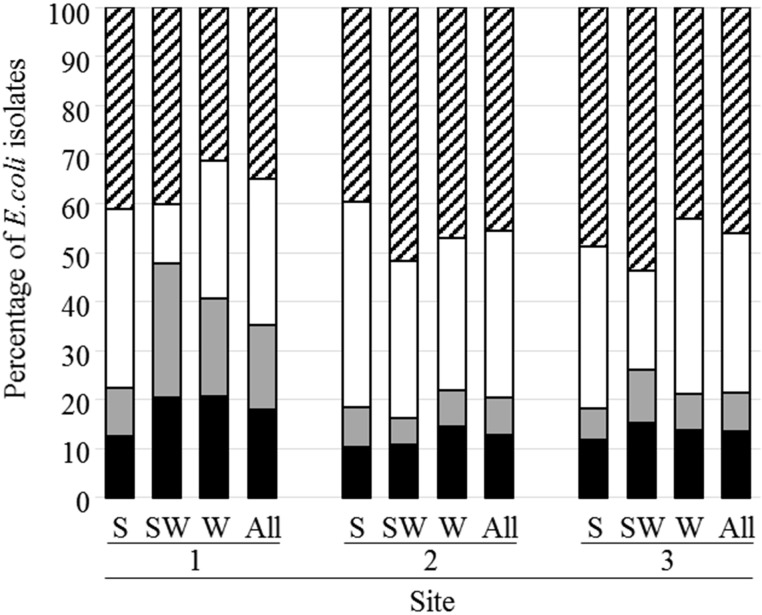
Distribution of *E. coli* isolates according to their phylogenetic group. The phylogenetic groups A, B1, B2, and D are represented by hatched, white, gray and black bars, respectively. S, shellfish; SW, seawater; W, river or brackish water.

7,452 *E. coli* strains were isolated and classified in four phylogenetic groups, 40.5, 31.5, 12.1, and 15.9% of strains belong to phylogroups A, B1, B2, and D, respectively (**Figure 3** and **Supplementary Data S4**). Two thousand and twenty four strains from phylogroups B2 and D, (1,146 from D and 876 from B2) were analyzed for the presence of nine virulence genes (*fyuA, hlyC, sfa, papC, iucC, papGIII, cnf1, papGII*, and *iroN*). This analysis (the complete results are presented in **Supplementary Data S4**) revealed a higher percentage of strains from site 1 carrying the *fyuA*, *sfa*, or *papGIII* genes compared to strains from the other sites. It also revealed that the mean virulence score was higher for strains belonging to B2 phylogroup (3.9) than for those of phylogroup D (3.0).

Four hundred and forty nine *E. coli* strains were analyzed to evaluate their level of resistance against a panel of 16 antibiotics or combinations of antibiotics (**Supplementary Data S4**). The results showed that a higher proportion of *E. coli* from Les Hardes river (sub-catchment of site 3) were resistant to cefoxitin, amoxicillin + clavulanic acid, and amoxicillin when compared to isolates from the other rivers. On the other hand, isolates from Le Frémur river (sub-catchment of site 1) were statistically more frequently resistant to doxycycline.

### Characterization of Enterococci

**FIGURE 4:**
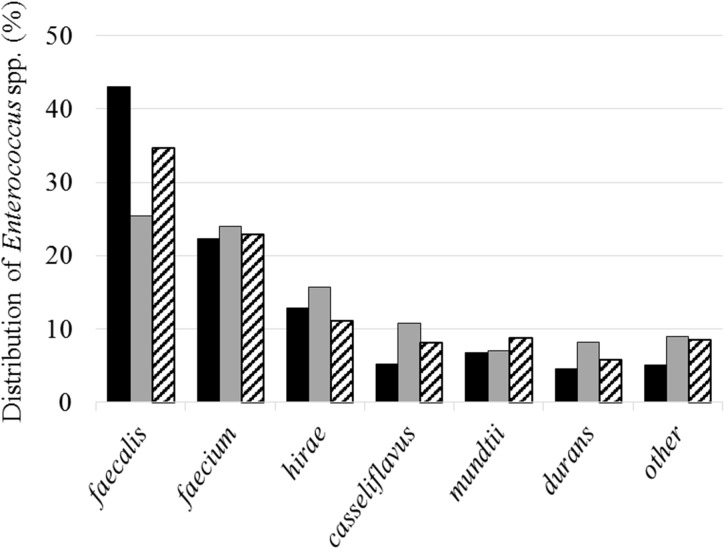
Distribution of the main isolated species. Black, gray and hatched bars represent results from sites 1, 2, and 3, respectively.

A total of 4,344 enterococcal strains were isolated, of which 3,887 were identified by mass spectrometry. These strains belong to 26 different species where *E. faecalis* (32.9%), *E. faecium* (23.2%), *E. hirae* (13.7%), *E. casseliflavus* (8.5%) *E. mundtii*, (7.4%), and *E. durans* (6.5%) represent the predominant species (**Figure 4**). The *H*′ biodiversity index of Shannon was similar for the three sites (2.5, 2.9, and 2.7, respectively, for sites 1, 2, and 3). These species were also the most abundant in water samples (*E. faecalis*: 29.1%, *E. faecium*: 23.6%, and *E. hirae*: 14.0%) and in shellfish (*E. faecalis*: 39.0%, *E. faecium*: 22.6%, and *E. hirae*: 12.9%).

Two thousand and sixty-nine strains (1,200 *E. faecalis* and 869 *E. faecium*) were analyzed for the presence of three virulence genes (*esp*, *gelE*, and *agg*) (**Supplementary Data S4**). These genes were more frequently detected within *E. faecalis* than *E. faecium* species. Their occurrence was comparable from one site to another, except for *gelE*, which was more frequently detected in *E. faecalis* from site 1 (56.3%) than from sites 2 and 3 (45.8%).

Susceptibility of 213 *E. faecalis* and 233 *E. faecium* strains to 16 antibiotics was also determined (**Supplementary Data S4**). Interestingly, the percentage of *E. faecium* resistant to levofloxacin, ampicillin, and streptomycin was higher for strains isolated from water samples than from shellfish. Analysis of isolates from rivers, revealed that 64.3% of *E. faecium* strains from La Sienne river (sub-catchment of site 2) and 55.5% from La Soules (sub-catchment of site 2) were resistant to ampicillin, whereas all strains from the other rivers were sensitive to this antibiotic. Furthermore, *E. faecium* strains isolated from La Sienne were also statistically more frequently resistant to levofloxacin.

### Salmonella

**FIGURE 5:**
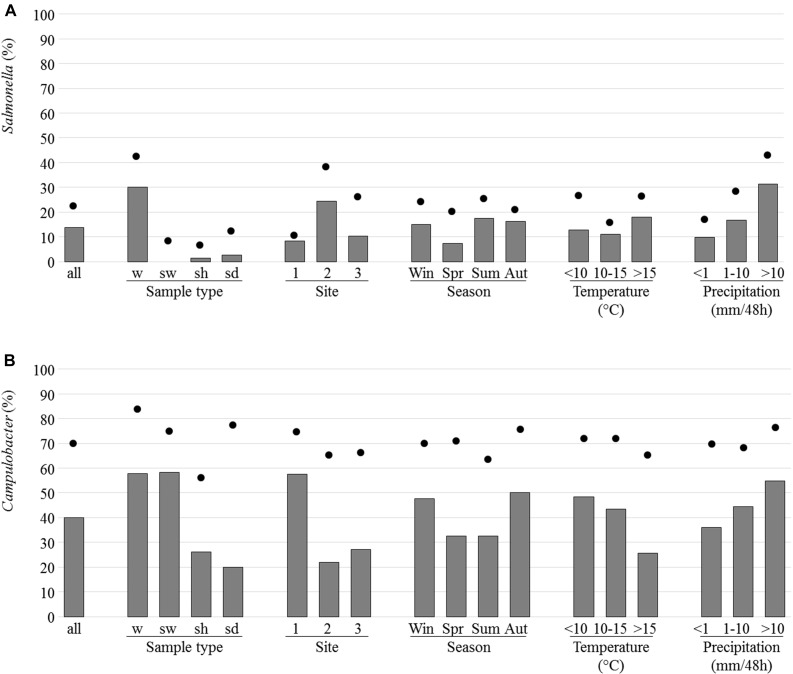
Prevalence and isolation of *Salmonella* **(A)** and *Campylobacter* **(B)**. The prevalence which corresponds to the presence of the *invA* or *ttrBCA* genes in selective enrichments for *Salmonella* or 16S RNA genes in Bolton broth for *Campylobacter* (dots) and the percentage of samples for which at least one strain was isolated (bars) are indicated as regard to sample type [water from river and brackish water (w), seawater (sw), shellfish (sh), and sediment (sd), site, season, temperature, and precipitation].

*Salmonella* spp. (468 colonies in total) were isolated from 69 samples (13.7% of the 505 samples tested). These samples were mainly from river and brackish waters (30.1% of sample with isolated *Salmonella*) and rarely from shellfish (3/237; 1.3%) or sediment (2.5%) while none of the 12 seawater samples analyzed allowed isolation of *Salmonella* (**Figure 5A**). The site 2 presented a higher percentage of samples with *Salmonella* (24.4%) and it was when the height of rainfall exceeded 10 mm per 48 h that the highest percentage of positive samples was observed (**Figure 5A**). 51.4% of the river and brackish water samples from site 2 and 19.8 and 18.7% of the corresponding samples from sites 1 and 3, respectively, were positive for *Salmonella s*pp. Positive samples were found at each of the four seasons (**Figure 5A**).

Of the 468 colonies, 75 *Salmonella* isolates were retained for further analyses. The remaining 393 isolates were considered replicates for the purpose of the present study (same serovar from the same sample or isolates from the same sample not serotyped). Seventy-one originated from water, three from shellfish (one from each type of shellfish and from each site) and one from sediment. These strains were assigned to *Salmonella enterica* subsp. *enterica* for 73 isolates and to *Salmonella enterica* subsp. *diarizonae* for two isolates belonging to the same 21:z10:z53 antigenic formulae. Among *S. enterica* subsp. *enterica*, the isolates were divided into 13 serovars (**Table 1**). Mbandaka was the most frequent serovar (36.0%) and was the only one found in the three positive shellfish batches. Eight strains from Typhimurium serovar were isolated (10.7%) and 10 others carrying O antigens O:4,5,12 were non-motile (two) or monophasic (eight) with antigen H:i, and one carrying O:4,12 antigen was monophasic with antigen H:i. CRISPOL typing confirmed that *Salmonella* Typhimurium and monophasic or non-motile variants belonged to the Typhimurium population of *Salmonella* and displayed diverse CRISPOL types (CT) 1, 18, 21, 30, 76, or 255. The CRISPOL types the most frequently found were CT 1 and 18 and mainly came from the Brittany site (three different rivers).

**Table 1:** Presence of *Salmonella* serovars and *Campylobacter* species in the three sites and in different types of samples (only isolates from different samples and different species from the same sample were retained).

	Site			Sample type			
Serovars/species	1	2	3	w	sw	sh	sd
***Salmonella* spp.**							
21:z10:z53:-	2			2			
Eboko		6	3	9			
Hessarek	1			1			
Kottbus		1	1	2			
Livingstone		1		1			
Mbandaka	2	21	4	24		3	
Menston		1		1			
Montevideo	3	4		7			
Rough		1		1			
Stourbridge	3	1	1	5			
Typhimurium	6	1	1	7			1
4,12:i:- (monophasic variant)			1	1			
4,5,12:-:- (non-motile)	2			2			
4,5,12:i:- (monophasic variant)	7	1		8			
**Total**	**26**	**38**	**11**	**71**	**0**	**3**	**1**
							
***Campylobacter* spp.**							
*C. coli*	59	21	13	82	3	7	1
*C. jejuni*	55	8	15	73	1	2	2
*C. lari*	71	9	1	23	6	57	4
*C. peloridis*	2	1	2	1		3	1
*C. upsaliensis*	1				1		
**Total**	**188**	**39**	**40**	**179**	**11**	**69**	**8**

*Sample type (water from river and brackish water (w), seawater (sw), shellfish (sh), and sediment (sd)].*

Several *Salmonella* serovars were isolated from each site and their distribution was different from one site to another. On the site 1, mainly *S.* Typhimurium and its variants were isolated whereas *S.* Mbandaka and, in a lesser extent, *S.* Eboko were mainly isolated on the sites 2 and 3 (**Table 1**). If *S.* Typhimurium, monophasic variants, *S.* Mbandaka, and *S.* Stourbridge were isolated at least once from all sites, the serovars Eboko or Kottbus and Montevideo were only isolated from sites 2 and 3, and sites 1 and 2, respectively. The other serovars were isolated once or twice from only one site. On the site 1, the *S.* Typhimurium and monophasic variants were mainly isolated from Le Rat sampling site.

Among 14 *S.* Mbandaka typed by PFGE, we found six different pulsotypes. WGS analysis performed on 21 *S.* Mbandaka indicated that all belonged to the MLST type ST413 and none antibiotic gene was found. On all of them we counted 1,296 SNPs and phylogenic analysis did not allowed us to detect any clusters under 50 SNP divergence, suggesting high diversity population and so absence of a unique source.

### Campylobacter

*Campylobacter* (1,400 colonies in total) were isolated from 204 of the 505 samples corresponding to 58.3% water, 26.6% shellfish and 20% sediment samples analyzed (**Figure 5B**). It was on site 1 and when rainfall height exceeded 10 mm per 48 h that the highest percentage of positive samples was observed (**Figure 5B**).

*Campylobacter jejuni* and *C. coli* were the most frequently found species in river and brackish water samples with 33.8 and 38% of samples positive for these species, respectively, versus 10.6 and 0.5% of samples positive for *C. lari* and for *C. peloridis*, respectively. *C. lari* was the most frequently isolated species in shellfish with 26.4% of samples positive for this species versus 0.8% for *C. jejuni*, 2.9% for *C. coli*, and 1.3% for *C. peloridis* (**Table 1**). More positive samples were observed in autumn and winter with temperature mainly under 15°C (**Figure 5B**). *C. jejuni* and *C. coli* were more frequently isolated in autumn (36.3% of positive samples) and *C. lari* in winter (34.6%). One species was isolated in 72.1% of the samples positive for *Campylobacter* spp. (147 of 204) whereas two species were isolated in 25% of the samples and only six samples (2.9%) allowed the isolation of three species.

Of the 1,400 clones, 267 *Campylobacter* isolates were retained for further analyses (isolates from different samples and different species from the same sample). One hundred and seventy nine originated from river and brackish waters, 11 from seawater, 69 from shellfish and eight from sediment. One hundred and eighty eight were isolated from site 1, 39 from site 2, and 40 from site 3.

*Campylobacter* spp. were more frequently isolated than *Salmonella* spp on these sites. In fact, 169 of the 505 samples (33.5%) were positive for *Campylobacter* spp. only versus 35 (6.9%) positive for *Salmonella* only and 34 (6.7%) positive for both pathogens.

### Vibrio

**FIGURE 6:**
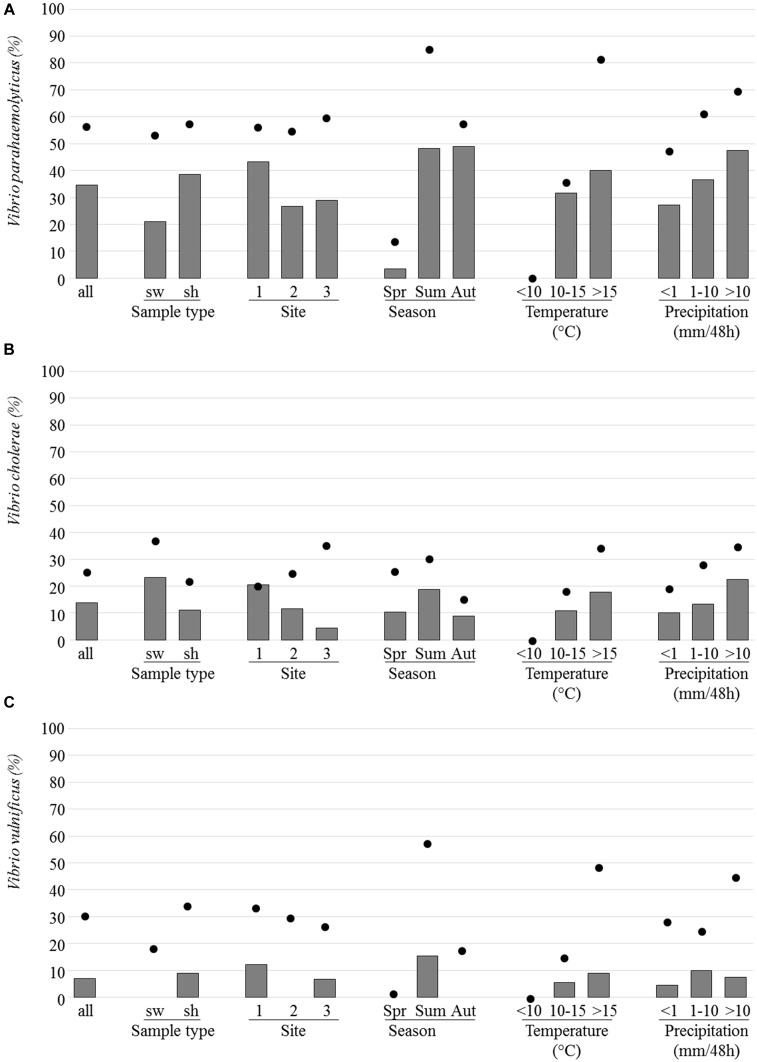
Prevalence and isolation of *Vibrio parahaemolyticus, Vp* **(A)**, *Vibrio cholerae, Vc* **(B)**, and *Vibrio vulnificus, Vv* **(C)**. The prevalence which corresponds to the presence of the *toxR* gene, the IGS region and the *vvhA* gene in selective enrichments for *Vp*, *Vc*, and *Vv*, respectively (dots) and the percentage of samples for which at least one strain was isolated (bars) are indicated as regard to sample type [seawater (sw), and shellfish (sh), site, season, temperature, and precipitation].

Among the 188 analyzed samples (43 seawaters and 145 shellfish batches), 56.9, 25.5, and 30.9% were positive for *V. parahaemolyticus*, *V. cholerae*, and *V. vulnificus*, respectively. For these *Vibrio* species, the highest prevalence was observed during the summer months and when water temperatures were above 15°C and/or when the height of precipitation exceeded 10 mm per 48 h (**Figure 6**). None of them was detected when water temperature was below 10°C.

*Vibrio parahaemolyticus* was the most frequently detected *Vibrio* species in the three sites with 56.6, 55.0, and 60.0% of positive samples from sites 1, 2, and 3, respectively. The prevalence of total *V. parahaemolyticus* was quite similar in seawater (53.5%) and shellfish (57.9%) (**Figure 6**). It was detected alone (39.2% of the 107 positive samples) or in combination with one (*V. vulnificus*, 29.0%; *V. cholerae*, 16.8%) or the two other species (15.0%). Enteropathogenic *tdh* positive *V. parahaemolyticus* was present in only six shellfish and two seawater samples and in the three sites but no *tdh* positive *Vp* strain was isolated. The prevalence of potentially pathogenic *trh* positive *V. parahaemolyticus* samples was higher in seawater than in shellfish samples (92.3 and 88.1%, respectively). It was detected in all the *V. parahaemolyticus* positive samples collected in Brittany (site 1). Trh strains were isolated from 58% of the *V. parahaemolyticus* positive samples.

*Vibrio cholerae* was found in seawater and shellfish (37.2 and 22.1%, respectively) and most prevalent in site 3 whereas *V. vulnificus* was detected more often in shellfish (34.5%) than in seawater samples (18.5%) and most prevalent in site 1 and during the summer months. *V. cholerae* strains were isolated from 13.8% of analyzed samples and *V. vulnificus* strains from 6.9%.

### Human Noroviruses

A total of 150 shellfish samples were analyzed for HuNoVs (**Table 2**). The average extraction efficiency was 9% and varied from 2% for cockle samples to 12% for mussel samples. HuNoVs were detected in 33 (22%) of the samples analyzed, 12 being under the limit of quantification (35 RNAc/g of DT). GM concentrations calculated for each species were comparable. As expected HuNoV positive samples were detected mainly during autumn and winter (**Table 3**). A similar rate of positive samples for HuNoV was observed between site 1 (21.1%) and site 2 (23.6%).

**Table 2:** HuNoV contamination in the shellfish species analyzed.

	Number of samples	Extraction efficiency (%)	HuNoV detection			
			Negative (<LD)	Positive (<LQ)	Positive	GMC
Cockles	30	2	25	1	4	117
Mussels	59	12	45	5	9	119
Oysters	61	10	47	6	8	140
Total	150	9	117	12	21	

*LD, limit of detection; LQ, limit of quantification; GMC, geometric mean concentration expressed as RNAc/g of digestive tissue*.

**Table 3:** Seasonal variation of HuNoV contamination.

	Spring	Summer	Autumn	Winter	Total
Number of samples	39	42	41	28	150
Extraction efficiency (%)	9	7	12	10	9
Number of positive samples (and %)	6	0	9	18	33
	(15.4%)	(0%)	(22.0%)	(64.2%)	(22.0%)

### Correlation Between Enteric Bacterial Pathogens, HuNoV and Concentration of *E. coli*

Comparison of results from isolation of enteric bacterial pathogens or detection of HuNoV with *E. coli* enumeration showed a positive correlation between the presence of *Salmonella* and *E. coli* concentration (OR = 3.94; **Table 4**). The relative risk of isolating *Salmonella* in samples whose *E. coli* concentration exceeds the median was 3.28 times higher than in those with *E. coli* concentration below the median. A positive correlation was also observed between the presence of STEC and/or EPEC and samples with the highest *E. coli* concentration (OR = 1.74 and RR = 1.57). In addition, a positive correlation between the presence of enteric pathogens was observed for *Salmonella* with *C. jejuni* and/or *C. coli* (OR = 2.58, RR = 2.21), for STEC and/or EPEC with *C. jejuni* and/or *C. coli* (OR = 2.79, RR = 2.00), and for *C. lari* with HuNoV (OR = 2.42, RR = 1.77).

**Table 4:** Correlations between *E. coli* concentration, isolation of pathogenic enteric bacteria and detection of HuNoVs.

	*C. jejuni* and/or *C. coli*		*C. lari*		STEC/EPEC		HuNoV		*E. coli*	
	+	-	+	-	+	-	+	-	>median^∗^	<median^∗^
Sample N°	130	375	90	415	89	416	33	117	231	233
*Salmonella* +	30 (23.1%)	39 (10.4%)	4 (4.4%)	65 (15.7%)	16 (18.0%)	53 (12.7%)	0 (0%)	1 (0.8%)	52 (76.5%)	16 (23.5%)
*Salmonella* -	100 (76.9%)	336 (89.6%)	86 (95.6%)	350 (84.3%)	73 (82.0%)	363 (87.3%)	33 (100%)	116 (99.2%)	179 (45.2%)	217 (54.8%)
OR	**2.58 *P* = 0.0004**		0.25 *P* = 0.0089		*1.50 P = 0.194*		*1.16 P = 0.928*		**3.94 *P* < 0.0001**	
RR	**2.21 *P* = 0.0003**		0.28 *P* = 0.0121		*1.41 P = 0.186*		*1.16 P = 0.928*		**3.28 *P* < 0.0001**	
*C. jejuni* and/or *C. coli*+			22 (24.4%)	108 (26.0%)	39 (43.8%)	91 (21.9%)	1 (3.0%)	5 (4.3%)	58 (45.0%)	71 (55.0%)
*C. jejuni* and/or *C. coli* -			68 (75.6%)	307 (74.0%)	50 (56.2%)	325 (78.1%)	32 (96.7%)	112 (95.7%)	173 (51.6%)	160 (48.4%)
OR			*0.92 P = 0.756*		**2.79 *P* < 0.0001**		*0.70 P = 0.749*		*0.755 P = 0.178*	
RR			*0.93 P = 0.757*		**2.00 *P* < 0.0001**		*0.71 P = 0.750*		*0.82 P = 0.179*	
*C. lari*+					14 (15.7%)	76 (18.3%)	15 (45.4%)	30 (25.6%)	43 (50.0%)	43 (50.0%)
*C. lari* -					75 (84.3%)	340 (81.7%)	18 (54.5%)	87 (74.4%)	188 (49.7%)	190 (50.3%)
OR					*0.83 P = 0.570*		**2.42 *P* = 0.0309**		*1.01 P = 0.964*	
RR					*0.86 P = 0.574*		**1.77 *P* = 0.0206**		*1.01 P = 0.965*	
STEC/EPEC+							2 (6.1%)	9 (7.7%)	53 (60.9%)	34 (39.1%)
STEC/EPEC -							31 (93.9%)	108 (92.3%)	178 (47.2%)	199 (52.8%)
OR							*0.77 P = 0.751*		**1.74 *P* = 0.022**	
RR							*0.78 P = 0.752*		**1.57 *P* = 0.0231**	
Sample N∘									74	76
HuNoV+									13 (17.6%)	20 (26.3%)
HuNoV -									61 (52.1%)	56 (47.9%)
OR									*0.60 P = 0.198*	
RR									*0.67 P = 0.202*	

*STEC/EPEC data were from Balière et al. (2015).*

*OR, odds ratio; RR, relative risk, data in bold: positive regulation, data in italics: P-value > 0.05.*

*^∗^Samples whose E. coli concentration is greater than, or less than, the median calculated by type of sample (shellfish, river or brackish waters, seawater).*

## Discussion

This original study aims to detect and characterize a large panel of micro-organisms from fecal contamination (*Salmonella* spp., *Campylobacte*r spp., *E. coli* and enterococci strains and HuNoVs) as well as some marine bacteria such as *Vibrio* spp.. For this study samples were collected from shellfish-harvesting areas and their catchments, but also from seawater. This has rarely been performed, most of other reporting data on these pathogens specifically relate either in shellfish or in water samples.

The enumerations of the FIB showed that water from all three catchments is characterized by fecal pollution that is variable and can reach relatively high levels at certain periods.

Classification of *E. coli* into phylogenetic groups revealed that isolates belonging to phylogroup A or B1 were more predominant than clones from group B2 or D. This observation is in agreement with previous results of surface water samples and shellfish (Stange et al., 2016; Vignaroli et al., 2016). Previous data obtained from the same river water samples showed that 5.6 and 21.3% of these waters were contaminated with STEC and EPEC, respectively (Balière et al., 2015). This means that these pathotypes were less common than other pathogenic bacteria mentioned above. Here, we focused on characterization of *E. coli* strains belonging to phylogroups B2 or D, the most frequently represented within ExPEC.

Analysis of antibiotic susceptibility of 338 *E. coli* isolated from river and brackish water samples and which belongs to phylogroups B2 or D showed a high proportion of bacteria resistant to cefoxitin, amoxicillin + clavulanic acid and amoxicillin in Les Hardes river and of clones resistant to doxycycline in Le Frémur.

Numerous studies have clearly shown that extraintestinal pathogenic strains generally contain more virulence factors than commensal strains. Here, we found that the average virulence score was higher for strains isolated from shellfish in site 1 than in sites 2 and 3. Recently, Johnson et al. (2017) assigned the ExPEC status to *E. coli* isolated from surface waters and animals based on the detection of a set of virulence genes. Their analysis focused on the detection of *papAH* and/or *papC* (encoding P fimbriae), *sfa*/*focDE* (S and F1 fimbriae), *afa*/*draDC* (Dr-binding adhesins), *kpsMI* (group 2 capsule) and *iutA* (aerobactin system); a strain being considered ExPEC when positive for at least two of these genes. In the present study, *papC* and *sfa* were selected and the percentage of strains isolated from shellfish that were positive for these two genes was also higher for site 1 (12.5%) than for sites 2 and 3 (8.5 and 9.3%, respectively). This would mean that the risk associated with the presence of ExPEC would be higher at the site 1.

In water, the predominant *Enterococcus* species were *E. faecalis*, *E. faecium*, and *E. hirae* followed by *E. casseliflavus*, *E. mundtii*, and *E. durans* which is in agreement with the species usually cited as the most abundantly detected in surface waters (Lanthier et al., 2011; Furtula et al., 2013; Sidhu et al., 2014; Veljović et al., 2015). As previously observed by Sidhu et al. (2014) virulence genes were detected more frequently in *E. faecalis* than in *E. faecium* isolates. For the collection of environmental *E. faecalis* tested here, 34.9, 49.8, and 46.0% of strains were positive for genes *esp*, *gelE*, and *agg*, respectively. These values, although high, are lower than those of clinical isolates described by Soares et al. (2014) who observed 70.1, 78.7, and 63.4% *E. faecalis* strains containing *esp*, *gelE*, and *agg* genes. As described by Lanthier et al. (2011), the presence of enterococci with multiple virulence genes is an additional risk factor for public health. Among the *E. faecalis* analyzed here, it is in the site 1 that we observed the highest percentage of isolates positive for at least two virulence genes.

Analysis of antibiotic susceptibility of *Enterococcus* isolates indicated that the sub-catchments of the rivers La Sienne and La Soules constitute the main source of ampicillin-resistant *E. faecium* and that most of the *E. faecium* isolates from the river La Sienne were resistant to levofloxacin. Additional analyses including the study of antibiotics used in farms or in human medicine on this particular site should make it possible to identify more precisely the corresponding source.

*Salmonella* was frequently detected in the three coastal catchments and *Salmonella* spp. was isolated in 30.1% of samples (range from 17.8% in site 1 to 51.4% in site 2). Such variable frequency of *Salmonella* positive samples has been already reported in the literature. Indeed, 43% of water samples from Georgia (United States) and 23% of surface water in Canada were also positive for *Salmonella* (Vereen et al., 2013; Flockhart et al., 2017).

A different distribution of serovars was observed according to the site. *S.* Typhimurium, and monophasic variants were mainly isolated on the site 1 whereas *S.* Mbandaka was mainly observed on site 2. This could be explained by the different agricultural activities on these catchments, with mainly swine, cattle and poultry breeding in Brittany and cattle, sheep and poultry in Normandy (**Supplementary Data S1**). *Salmonella* Mbandaka are known to be mainly present in cattle and poultry (European Food Safety Authority [EFSA] and European Centre for Disease Prevention Control [ECDC], 2015) while swine are one of the major sources of *Salmonella* Typhimurium, in particular its monophasic variants (Martelli et al., 2018). Furthermore, the main CRISPOL type identified in these latter strains was the CT1, a *Salmonella* population known to be responsible for most of human infections and frequently abundant in swine in France (Fabre et al., 2012). *S.* Mbandaka was described as highly persistent in the environment near farms due to its ability to produce biofilms and to persist and grow in the external environment and on animal feed (Hayward et al., 2016). Furthermore, *S.* Mbandaka was found to be more resistant to sunlight (solar simulator; passing wavelength, 290 nm < λ < 800 nm) than *S.* Typhimurium and other serovars (Boehm et al., 2012). Contrary to *S.* Mbandaka, *S.* Typhimurium and monovariants are one of the serotypes frequently implicated in food-borne outbreaks (European Food Safety Authority [EFSA] and European Centre for Disease Prevention Control [ECDC], 2016). The main presence of *S.* Typhimurium in water samples was already observed in coastal catchments in United States and catchments in Canada with also the presence of *S.* Mbandaka (Walters et al., 2013; Jokinen et al., 2015).

In shellfish, the prevalence of *Salmonella* spp. was low (1.3%) and similar to those observed in market oysters in United States (DePaola et al., 2010) but lower than those from other studies (8% of molluscs in Northern Ireland, Wilson and Moore, 1996; 7.4% of oysters from 36 United States bays, Brands et al., 2005; 10% of mussels in Morocco; Setti et al., 2009). Interestingly, the presence of only one serovar that is *S.* Mbandaka (three shellfish batches in the three sites) was different from previous studies in which several serovars such as *S.* Newport, *S.* Typhimurium, *S.* Agona, *S.* Blockey, *S.* Kentucky were isolated (Brands et al., 2005; Setti et al., 2009). The presence of only *S*. Mbandaka in shellfish could be explained by the better survival of this serovar in the environment and the presence of poultry and cattle breeding, main reservoir of *S*. Mbandaka, upstream the shellfish-harvesting areas. The identification of this serovar suggests a low risk of contamination by *Salmonella* in shellfish. However, *S.* Mbandaka ST413 (the main ST of this serovar and found in all isolates here) was responsible of food-borne illnesses in Poland (Hoszowski et al., 2016).

As for *Salmonella*, *Campylobacter* has been frequently isolated in the studied catchments, with an isolation in 58.3% of water samples. On the three sites, water samples were more frequently positive for *C. coli* and *C. jejuni* than *C. lari*. The highest prevalence of *C. jejuni* and *C. coli* was also observed by Denis et al. (2011b) and Khan et al. (2014) who found more frequently *C. jejuni* than *C. coli* and *C. lari* in Canada and in France (Brittany), respectively. These different species could arise from both human and animal origins (Pitkanen and Hanninen, 2017). If we consider the breeding sites and the prevalence described for *C. coli*, *C. jejuni*, and *C. lari* in the feces and manure of the corresponding animals, *C. coli* could arise mainly from swine, poultry, and sheep and *C. jejuni* from cattle, poultry and sheep. Furthermore, another important source of *Campylobacte*r, including *C. lari* could be wild birds (Ryu et al., 2014).

In shellfish, the prevalence of *C. jejuni* (0.8%) and *C. coli* (2.9%) was very low whereas *C. lari* was most frequently isolated in these samples (24.1%) than in river waters. *Campylobacter* was the most often bacterial enteric pathogen isolated in shellfish (27.8% of samples positive for *Campylobacter*) which is lower than the 42% of shellfish with thermophilic *Campylobacter* spp. observed by Wilson and Moore (1996). Interestingly, a similar distribution among the species was observed in both studies. Indeed, *C. jejuni, C. coli*, and *C. lari* represented 3, 10.6, and 86.1% of the *Campylobacter* spp. isolated in the present study, respectively, and 2, 8, and 81% of the *Campylobacter* spp. isolated by Wilson and Moore, respectively. The fact that *C. lari* was more present in marine environment and especially in shellfish comparing to *C. jejuni* and *C. coli*, whereas the opposite was observed in upstream waters, suggests a better persistence of this species in marine environment and/or an input from wild seabirds, as observed previously in Morecambe Bay (United Kingdom) by Obiri-Danso et al. (2001). These two hypotheses will be investigated in the future.

Concerning the marine bacteria, the incidence of total *Vibrio parahaemolyticus* was similar in shellfish and seawater samples (57.9 and 53.5%) and equally distributed between the three sites (55–60%). Culturable *V. parahaemolyticus* was detected in summer and autumn although the highest incidence (85.9%) was found in summer. Previous studies conducted in European coastal areas showed lower occurrence of *V. parahaemolyticus* in seawaters (12–31.4%) (Deter et al., 2010; Rodriguez-Castro et al., 2010; Martinez-Urtaza et al., 2012). However, similar or higher prevalence of *V. parahaemolyticus* was recorded in shellfish samples analyzed over the same seasonal period (Collin and Rehnstam-Holm, 2011; Suffredini et al., 2014).

The first incidence of *V. parahaemolyticus tdh* gene in Northern French waters confirm the recent reports of the presence of *V. parahaemolyticus* carrying this gene in Northern European waters (Collin and Rehnstam-Holm, 2011; Powell et al., 2013). However, no *tdh*+ strain was isolated in any of the three costal sites. The prevalence of *V. parahaemolyticus trh* gene in the environment appears very high (88.8%) and contrasts with previous observations in Southern and Northern Europe (Hervio-Heath et al., 2002; Esteves et al., 2015; Passalacqua et al., 2016).

*Vibrio vulnificus* and *V. cholerae* non-O1/non-O139 were detected in 30.9 and 25.5% of the environmental samples (34.5 and 22.2% in shellfish and 18.6 and 37.2% in seawater, respectively). This occurrence is much higher than previously reported in Europe for environmental samples except for blue mussels collected in 2006 in The Sound between Denmark and Sweden (63 and 53%, respectively) (Collin and Rehnstam-Holm, 2011). Recent emergence of wound and otitis infections caused by these two *Vibrio* species in Northern Europe (North Sea and Baltic Sea region) coincided with warm weather anomalies during summer months (Baker-Austin et al., 2013). These observations have to be considered and suggest that the presence of *V. cholerae* and *V. vulnificus* in French coastal waters could pose a potential hazard to shellfish consumers and for susceptible people exposed to the seawater especially during the summer months.

For HuNoVs the percentage of positive samples was higher compared to other studies performed in our country confirming the fecal contamination shown by FIB counts (Schaeffer et al., 2013). Extraction efficiency was always above the quality criteria stipulated in the ISO method, and is similar to results from previous studies (Schaeffer et al., 2013, 2018; Le Mennec et al., 2017). Even if most of samples presented a low concentration, this can be an issue for marketing considering the high infectivity of these viruses (Polo et al., 2016). As expected, most of positive samples were detected during the cold season when the virus is circulating in the human population and thus excreted in sewage (de Graaf et al., 2016).

The seasonal influence on the presence of human potential pathogens in shellfish was also observed for other human potential pathogens in this study. Firstly, as discussed above, *Vibrio* spp. were mainly detected/isolated on summer at the opposite to HuNoVs mainly detected in winter.

Concerning bacterial enteric pathogens, human *Salmonella* and *Campylobacter* infections typically occur in summer although environmental studies often show varied seasonal peaks for these pathogens (Vereen et al., 2013). Here, *Campylobacter jejuni, C. coli*, and *C. lari* were detected all year round in the environmental samples. However, a seasonal effect was observed with more positive samples in autumn and winter. *C. jejuni* and *C. coli* were more frequently isolated in autumn (36.3% of positive samples) whereas *C. lari* was more frequently isolated in winter (34.6%). These results are in agreement with those previously obtained in river water (Llobregat river, Spain), freshwater bathing sites (River Lune, United Kingdom) and coastal areas (Morecambe Bay and Lune Estuary, United Kingdom) which have shown higher *Campylobacter* concentrations in winter than in summer (Obiri-Danso and Jones, 1999; Jones, 2001; Rodriguez and Araujo, 2012). Vereen et al. (2013) also observed lower detection frequencies of *Campylobacter* in water of the Satilla River Basin (United States) in summer, reflecting a reverse association with temperature. Furthermore, a lower survival rate of *Campylobacter* in surface water in presence of sunlight (elevated UV levels) and higher temperatures has been observed (Obiri-Danso et al., 2001; Rodriguez and Araujo, 2012). In contrast, we did not show seasonal variation for *Salmonella* with year-round detection in rivers and only three isolations of *Salmonella* in shellfish (one in March and two in August).

*Salmonella*, *Campylobacter*, and *Vibrio* were more often detected after rainfall events in the three sites. This was also the case for *E. coli* concentrations (data not shown). For the enteric pathogens, these higher frequencies could be due to a higher release of these bacteria from wastes disposal in the fields by runoff and/or an exceedance of the waste water treatment plant capacity of collective plants, individual septic systems and/or non-collective sanitation. Rainfall was found to be the most significant environmental parameter driving the transfer of fecal contamination from soil to streams in three catchments in Brittany (including the site 1). In this later site, human-, bovine- and pig-associated markers of source of fecal contamination were more detected when rainfall >10 mm (Jardé et al., 2018).

For *Vibrio*, an input of nutrients to coastal waters by runoff could be in favor of a higher growth of these bacteria.

A variable presence of human potential pathogens according to the site was observed here for several pathogens. Firstly, *Campylobacter* was more frequently isolated in Brittany (site 1) than in Normandy (sites 2 and 3). Furthermore, *S.* Typhimurium and monophasic variants were the most frequently isolated *Salmonella* on the site 1 whereas it was *S.* Mbandaka on the site 2. These differences could be due to the urban and agricultural activities which are different in the studied sites and to the respective size of these catchments. If the variable densities of the different types of livestock (bovine, poultry, sheep, and pig) could explain the difference from one site to another, it is less clear at the level of sub-catchments. This lack of clear relationship in sub-catchments could be due to animal waste disposal practices. For example, in Brittany (site 1), poultry and pig were landless breeded whereas cattle are both reared outdoors on grass and in farms. These different rearing methods produce different types of wastes in this catchment which could be carried out both inside and outside the sub-catchment (pig manure), could be exported (poultry manure) or directly input on the field (cowpats).

Correlations were observed between the isolation of enteric bacterial pathogens and/or *E. coli* concentrations and likely reflect variations in waste disposal and runoff. In the selection of shellfish samples for which noroviruses were studied (*n* = 150), no correlation was found between the detection levels of *E. coli* or enteric bacteria *Salmonella*, STEC/EPEC or *C. jejuni* and/or *C. coli*, and HuNoVs whose circulation, as mentioned above, is more important in winter. On the other hand, a correlation was observed between HuNoVs and *C. lari* and this could be due to a similar seasonal distribution *of C. lari* and HuNoVs. To the best of our knowledge norovirus and bacteria co-infection in humans has not been reported yet. However, the discovery that human norovirus binds to some enteric bacteria raised hypothesis on the impact for their resistance in the environment, or to heat stress (Miura et al., 2013; Li et al., 2015) or may also play a role during infection (Sullender and Baldridge, 2018).

## Conclusion

We demonstrate a high prevalence of potential enteric bacterial pathogens in these coastal catchments but a low prevalence of *Salmonella* and *C. jejuni* and *C. coli* in shellfish from the downstream harvesting areas. No correlation between detection levels of *E. coli* and that of HuNoVs neither *Campylobacter* spp. was shown, at the opposite to *Salmonella.* However, more importantly, a positive correlation between the presence of *Salmonella* and *C. jejuni* and/or *C. coli* and between the presence of *C. lari* and HuNoVs was observed; this latter observation could be due to their similar season distributions or to other factors that need to be investigated. The different distribution of *Campylobacter* species in rivers and shellfish could be in favor of additional inputs of bacteria such as wild birds.

## Author Contributions

MG and AR designed the study, participated in the bacterial analysis, and wrote the manuscript. CB participated in the bacterial analysis and especially in the isolation of *E. coli* and enterococci. J-CG performed the characterization of *E. coli* and enterococci. DH-H participated in the *Vibrio* analysis and in manuscript writing. SL and JC isolated and analyzed *Salmonella* and *Campylobacter*, respectively. SLH characterized *Salmonella* (serotyping and WGS) and revised the manuscript. NS and AB contributed to the isolation and the analysis of bacteria. SP and SS performed the HuNoVs analysis. FLG analyzed the HuNoVs data and participated in the writing of the manuscript.

## Conflict of Interest Statement

The authors declare that the research was conducted in the absence of any commercial or financial relationships that could be construed as a potential conflict of interest.

## Footnotes

1 http://www.fao.org/food/food-safety-quality/scientific-advice/jemra/risk-assessments/vibrio0/en/

2 http://www.genomicepidemiology.org/

3 http://enterobase.warwick.ac.uk/

4 https://www.alyoung.com/labs/biodiversity_calculator.html

5 https://www.medcalc.org/calc/

## References

[B1] Abeyta C. Deeter F. G. Kaysner C. A. Stott R. F. Wekell M. M. (1993). *Campylobacter jejuni* in a Washington state shellfish growing bed associated with illness. *J. Food Prot.* 56 323–325. 10.4315/0362-028X-56.4.323 31091626

[B2] AFNOR-NF-V-08-106:2010 (2010). Microbiologie des aliments: Dnombrement des *E. coli* présumés dans les coquillages vivants -Technique indirecte par impédancemétrie directe.

[B3] Amagliani G. Brandi G. Schiavano G. F. (2012). Incidence and role of *Salmonella* in seafood safety. *Food Res. Int.* 45 780–788. 10.1016/j.foodres.2011.06.022

[B4] Andrade V. C. Zampieri B. B. Ballesteros E. R. Pinto A. B. de Oliveira A. J. (2015). Densities and antimicrobial resistance of *Escherichia coli* isolated from marine waters and beach sands. *Environ. Monit. Assess.* 187:342. 10.1007/s10661-015-4573-8 25963763

[B5] Atmar R. L. Opekun A. R. Gilger M. A. Estes M. K. Crawford S. E. Neill F. H. (2014). Determination of the 50% human infectious dose for Norwalk virus. *J. Infect. Dis.* 209 1016–1022. 10.1093/infdis/jit620 24253285 PMC3952671

[B6] Baker-Austin C. Trinanes J. A. Taylor N. G. H. Hartnell R. Siitonen A. Martinez-Urtaza J. (2013). Emerging *Vibrio* risk at high latitudes in response to ocean warming. *Nature Climate Change* 3 73–77. 10.1038/nclimate1628

[B7] Balière C. Rincé A. Blanco J. Dahbi G. Harel J. Vogeleer P. (2015). Prevalence and characterization of shiga toxin-producing and Enteropathogenic *Escherichia coli* in shellfish-harvesting areas and their watersheds. *Front. Microbiol.* 6:1356. 10.3389/fmicb.2015.01356 26648928 PMC4664706

[B8] Boehm A. B. Sassoubre L. M. (2014). “Enterococci as indicators of environmental fecal contamination,” in *Enterococci: From Commensals to Leading Causes of Drug Resistant Infection*, eds Gilmore M. S. Clewell D. B. Ike Y. Shankar N. (Boston, MA: Massachusetts Eye and Ear Infirmary). 24649503

[B9] Boehm A. B. Soetjipto C. Wang D. (2012). Solar inactivation of four *Salmonella* serovars in fresh and marine waters. *J. Water Health* 10 504–510. 10.2166/wh.2012.084 23165707

[B10] Bonacorsi S. Houdouin V. Mariani-Kurkdjian P. Mahjoub-Messai F. Bingen E. (2006). Comparative prevalence of virulence factors in *Escherichia coli* causing urinary tract infection in male infants with and without bacteremia. *J. Clin. Microbiol.* 44 1156–1158. 10.1128/JCM.44.3.1156-1158.2006 16517919 PMC1393126

[B11] Brands D. A. Inman A. E. Gerba C. P. Maré C. J. Billington S. J. Saif L. A. (2005). Prevalence of *Salmonella* spp. in oysters in the United States. *Appl. Environ. Microbiol.* 71 893–897. 10.1128/AEM.71.2.893-897.2005 15691945 PMC546685

[B12] Clermont O. Bonacorsi S. Bingen E. (2000). Rapid and simple determination of the *Escherichia coli* phylogenetic group. *Appl. Environ. Microbiol.* 66 4555–4558. 10.1128/AEM.66.10.4555-4558.2000 11010916 PMC92342

[B13] Collin B. Rehnstam-Holm A.-S. (2011). Occurrence and potential pathogenesis of *Vibrio cholerae*, *Vibrio parahaemolyticus* and *Vibrio vulnificus* on the South coast of Sweden. *FEMS Microbiol. Ecol.* 78 306–313. 10.1111/j.1574-6941.2011.01157.x 21692819

[B14] David J. M. Sanders P. Bemrah N. Granier S. A. Denis M. Weill F.-X. (2013). Attribution of the French human Salmonellosis cases to the main food-sources according to the type of surveillance data. *Prev. Vet. Med.* 110 12–27. 10.1016/j.prevetmed.2013.02.002 23453456

[B15] de Graaf M. van Beek J. Koopmans M. P. G. (2016). Human norovirus transmission and evolution in a changing world. *Nat. Rev. Microbiol.* 14 421–433. 10.1038/nrmicro.2016.48 27211790

[B16] Denis M. Henrique E. Chidaine B. Tircot A. Bougeard S. Fravalo P. (2011a). Campylobacter from sows in farrow-to-finish pig farms: risk indicators and genetic diversity. *Vet. Microbiol.* 154 163–170. 10.1016/j.vetmic.2011.07.001 21802224

[B17] Denis M. Tanguy M. Chidaine B. Laisney M.-J. Mégraud F. Fravalo P. (2011b). Description and sources of contamination by *Campylobacter* spp. of river water destined for human consumption in Brittany, France. *Pathologie Biologie* 59 256–263. 10.1016/j.patbio.2009.10.007 19942377

[B18] DePaola A. Jones J. L. Woods J. Burkhardt W. Calci K. R. Krantz J. A. (2010). Bacterial and viral pathogens in live oysters: 2007 United States market survey. *Appl. Environ. Microbiol.* 76 2754–2768. 10.1128/AEM.02590-09 20190085 PMC2863423

[B19] Deter J. Lozach S. Derrien A. Véron A. Chollet J. Hervio-Heath D. (2010). Chlorophyll a might structure a community of potentially pathogenic culturable *Vibrionaceae*. Insights from a one-year study of water and mussels surveyed on the French Atlantic coast. *Environ. Microbiol. Rep.* 2 185–191. 10.1111/j.1758-2229.2010.00133.x 23766015

[B20] Escobedo-Hinojosa W. Pardo-López L. (2017). Analysis of bacterial metagenomes from the Southwestern Gulf of Mexico for pathogens detection. *Pathog. Dis.* 75:ftx058. 10.1093/femspd/ftx058 28535299

[B21] Esteves K. Hervio-Heath D. Mosser T. Rodier C. Tournoud M.-G. Jumas-Bilak E. (2015). Rapid proliferation of *Vibrio parahaemolyticus*, *Vibrio vulnificus*, and *Vibrio cholerae* during freshwater flash floods in French Mediterranean coastal lagoons. *Appl. Environ. Microbiol.* 81 7600–7609. 10.1128/AEM.01848-15 26319881 PMC4592872

[B22] European Food Safety Authority [EFSA] and European Centre for Disease Prevention Control [ECDC] (2015). The European Union summary report on trends and sources of zoonoses, zoonotic agents and food-borne outbreaks in 2014. *EFSA J.* 13 1–190. 10.2903/j.efsa.2015.4329

[B23] European Food Safety Authority [EFSA] and European Centre for Disease Prevention Control [ECDC] (2016). The European Union summary report on trends and sources of zoonoses, zoonotic agents and food-borne outbreaks in 2015. *EFSA J.* 14 1–231. 10.2903/j.efsa.2016.4634 32625371 PMC7009962

[B24] Fabre L. Zhang J. Guigon G. Le Hello S. Guibert V. Accou-Demartin M. (2012). CRISPR typing and subtyping for improved laboratory surveillance of *Salmonella* infections. *PLoS One* 7:e36995. 10.1371/journal.pone.0036995 22623967 PMC3356390

[B25] Flockhart L. Pintar K. Cook A. McEwen S. Friendship R. Kelton D. (2017). Distribution of *Salmonella* in humans, production animal operations and a watershed in a FoodNet Canada Sentinel Site. *Zoonoses Public Health* 64 41–52. 10.1111/zph.12281 27345363

[B26] Fonteneau L. Jourdan Da Silva N. Fabre L. Ashton P. Torpdahl M. Müller L. (2017). Multinational outbreak of travel-related Salmonella Chester infections in Europe, summers 2014 and 2015. *Euro Surveill.* 22:30463. 10.2807/1560-7917.ES.2017.22.7.30463 28230522 PMC5322187

[B27] Furtula V. Jackson C. R. Farrell E. G. Barrett J. B. Hiott L. M. Chambers P. A. (2013). Antimicrobial resistance in *Enterococcus* spp. isolated from environmental samples in an area of intensive poultry production. *Int. J. Environ. Res. Public Health* 10 1020–1036. 10.3390/ijerph10031020 23481592 PMC3709301

[B28] Gorski L. Parker C. T. Liang A. Cooley M. B. Jay-Russell M. T. Gordus A. G. (2011). Prevalence, distribution, and diversity of *Salmonella enterica* in a major produce region of California. *Appl. Environ. Microbiol.* 77 2734–2748. 10.1128/AEM.02321-10 21378057 PMC3126348

[B29] Grimont P. A. D. Weill F. X. (2007). *Antigenic Formulae of the Salmonella serovars*, 9th Edn. Paris: WHO Collaborating Center for Reference and Research on Salmonella.

[B30] Guzman Prieto A. M. van Schaik W. Rogers M. R. C. Coque T. M. Baquero F. Corander J. (2016). Global emergence and dissemination of *Enterococci* as nosocomial pathogens: attack of the Clones? *Front. Microbiol.* 7:788. 10.3389/fmicb.2016.00788 27303380 PMC4880559

[B31] Hayward M. R. Petrovska L. Jansen V. A. A. Woodward M. J. (2016). Population structure and associated phenotypes of *Salmonella enterica* serovars Derby and Mbandaka overlap with host range. *BMC Microbiol.* 16:15. 10.1186/s12866-016-0628-4 26846255 PMC4743429

[B32] Hervio-Heath D. Colwell R. R. Derrien A. Robert-Pillot A. Fournier J. M. Pommepuy M. (2002). Occurrence of pathogenic vibrios in coastal areas of France. *J. Appl. Microbiol.* 92 1123–1135. 10.1046/j.1365-2672.2002.01663.x 12010553

[B33] Hoszowski A. Zająac M. Lalak A. Przemyk P. Wasyl D. (2016). Fifteen years of successful spread of *Salmonella enterica* serovar Mbandaka clone ST413 in Poland and its public health consequences. *Ann. Agric. Environ. Med.* 23 237–241. 10.5604/12321966.1203883 27294625

[B34] ISO-7899-1:1998 (1998). *Qualité de l’eau: Recherche et dénombrement des entérocoques intestinaux -Partie 1: Méthode miniaturisée (nombre le plus probable) pour les eaux de surface et résiduaires*. Available at: https://www.iso.org/fr/standard/14852.html

[B35] ISO-9308-3:1998 (1998). *Qualité de l’eau: Recherche et dénombrement des Escherichia coli et des bactéries coliformes -Partie 3: Méthode miniaturisée (nombre le plus probable) pour la recherche et le dénombrement des E. coli dans les eaux de surface et résiduaires*. Available at: https://www.iso.org/fr/standard/20878.html

[B36] Jardé E. Jeanneau L. Harrault L. Quenot E. Solecki O. Petitjean P. (2018). Application of a microbial source tracking based on bacterial and chemical markers in headwater and coastal catchments. *Sci. Total Environ.* 61 55–63. 10.1016/j.scitotenv.2017.07.235 28802110

[B37] Johnson J. R. Delavari P. Kuskowski M. Stell A. L. (2001). Phylogenetic distribution of extraintestinal virulence-associated traits in *Escherichia coli*. *J. Infect. Dis.* 183 78–88. 10.1086/317656 11106538

[B38] Johnson J. R. Porter S. B. Johnston B. Thuras P. Clock S. Crupain M. (2017). Extraintestinal pathogenic and antimicrobial-resistant *Escherichia coli*, including sequence type 131 (ST131), from retail chicken breasts in the United States in 2013. *Appl. Environ. Microbiol.* 83:e02956-16. 10.1128/AEM.02956-16 28062464 PMC5335533

[B39] Jokinen C. C. Koot J. Cole L. Desruisseau A. Edge T. A. Khan I. U. H. (2015). The distribution of *Salmonella enterica* serovars and subtypes in surface water from five agricultural regions across Canada. *Water Res.* 76 120–131. 10.1016/j.watres.2015.02.038 25799976

[B40] Jones K. (2001). Campylobacters in water, sewage and the environment. *Symp. Ser. Soc. Appl. Microbiol.* 30 68S–79S. 10.1046/j.1365-2672.2001.01355.x 11422562

[B41] Kaper J. B. Nataro J. P. Mobley H. L. (2004). Pathogenic *Escherichia coli*. *Nat. Rev. Microbiol.* 2 123–140. 10.1038/nrmicro818 15040260

[B42] Khan I. U. Gannon V. Jokinen C. C. Kent R. Koning W. Lapen D. R. (2014). A national investigation of the prevalence and diversity of thermophilic *Campylobacter* species in agricultural watersheds in Canada. *Water Res.* 61 243–252. 10.1016/j.watres.2014.05.027 24930011

[B43] Kreft B. Marre R. Schramm U. Wirth R. (1992). Aggregation substance of *Enterococcus faecalis* mediates adhesion to cultured renal tubular cells. *Infect. Immun.* 60 25–30. 1729187 10.1128/iai.60.1.25-30.1992 PMC257498

[B44] Lanthier M. Scott A. Zhang Y. Cloutier M. Durie D. Henderson V. C. (2011). Distribution of selected virulence genes and antibiotic resistance in *Enterococcus* species isolated from the South Nation River drainage basin, Ontario, Canada. *J. Appl. Microbiol.* 110 407–421. 10.1111/j.1365-2672.2010.04893.x 21091592

[B45] Le Mennec C. Parnaudeau S. Rumebe M. Le Saux J.-C. Piquet J.-C. Le Guyader F. S. (2017). Follow-up of norovirus contamination in an oyster production area linked to repeated outbreaks. *Food Environ. Virol.* 9 54–61. 10.1007/s12560-016-9260-6 27613529

[B46] Leblanc-Maridor M. Beaudeau F. Seegers H. Denis M. Belloc C. (2011). Rapid identification and quantification of Campylobacter coli and *Campylobacter jejuni* by real-time PCR in pure cultures and in complex samples. *BMC Microbiol.* 11:113. 10.1186/1471-2180-11-113 21600037 PMC3123193

[B47] Leight A. K. Crump B. C. Hood R. R. (2018). Assessment of fecal indicator bacteria and potential pathogen co-occurrence at a shellfish growing area. *Front. Microbiol.* 9:384. 10.3389/fmicb.2018.00384 29593669 PMC5861211

[B48] Li D. Breiman A. le Pendu J. Uyttendaele M. (2015). Binding to histo-blood group antigen-expressing bacteria protects human norovirus from acute heat stress. *Front. Microbiol.* 6:659. 10.3389/fmicb.2015.00659 26191052 PMC4486850

[B49] Martelli F. Andres V. M. Davies R. Smith R. P. (2018). Observations on the introduction and dissemination of *Salmonella* in three previously low prevalence status pig farms in the United Kingdom. *Food Microbiol.* 71 129–134. 10.1016/j.fm.2017.05.004 29366462

[B50] Martinez-Urtaza J. Blanco-Abad V. Rodriguez-Castro A. Ansede-Bermejo J. Miranda A. Rodriguez-Alvarez M. X. (2012). Ecological determinants of the occurrence and dynamics of *Vibrio parahaemolyticus* in offshore areas. *ISME J.* 6 994–1006. 10.1038/ismej.2011.156 22094349 PMC3329108

[B51] Martinez-Urtaza J. Saco M. de Novoa J. Perez-Piñeiro P. Peiteado J. Lozano-Leon A. (2004). Influence of environmental factors and human activity on the presence of *Salmonella* serovars in a marine environment. *Appl. Environ. Microbiol.* 70 2089–2097. 10.1128/AEM.70.4.2089-2097.2004 15066800 PMC383045

[B52] Miller W. G. Yee E. Chapman M. H. Smith T. P. L. Bono J. L. Huynh S. (2014). Comparative genomics of the *Campylobacter lari* group. *Genome Biol. Evol.* 6 3252–3266. 10.1093/gbe/evu249 25381664 PMC4986449

[B53] Miura T. Sano D. Suenaga A. Yoshimura T. Fuzawa M. Nakagomi T. (2013). Histo-blood group antigen-like substances of human enteric bacteria as specific adsorbents for human noroviruses. *J. Virol.* 87 9441–9451. 10.1128/JVI.01060-13 23804639 PMC3754087

[B54] Miura T. Schaeffer J. Le Saux J.-C. Le Mehaute P. Le Guyader F. S. (2017). Virus type-specific removal in a full-scale membrane bioreactor treatment process. *Food Environ. Virol.* 10 176–186. 10.1007/s12560-017-9330-4 29214559

[B55] Obiri-Danso K. Jones K. (1999). Distribution and seasonality of microbial indicators and thermophilic campylobacters in two freshwater bathing sites on the River Lune in northwest England. *J. Appl. Microbiol.* 87 822–832. 10.1046/j.1365-2672.2001.01239.x 10664907

[B56] Obiri-Danso K. Paul N. Jones K. (2001). The effects of UVB and temperature on the survival of natural populations and pure cultures of *Campylobacter jejuni*, Camp. *coli, Camp. lari* and urease-positive thermophilic campylobacters (UPTC) in surface waters. *J. Appl. Microbiol.* 90 256–267. 10.4081/ijfs.2016.5709 11168729

[B57] Passalacqua P. L. Zavatta E. Bignami G. Serraino A. Serratore P. (2016). Occurrence of *Vibrio parahaemolyticus*, *Vibrio cholerae* and *Vibrio vulnificus* in the clam Ruditapes Philippinarum (Adams & Reeve, 1850) from Emilia Romagna and Sardinia, Italy. *Ital. J. Food Saf.* 5:5709. 10.4081/ijfs.2016.5709 27800436 PMC5076712

[B58] Picard B. Garcia J. S. Gouriou S. Duriez P. Brahimi N. Bingen E. (1999). The link between phylogeny and virulence in *Escherichia coli* extraintestinal infection. *Infect. Immun.* 67 546–553. 9916057 10.1128/iai.67.2.546-553.1999 PMC96353

[B59] Pitkanen T. Hanninen M. L. (2017). *Members of the family Campylobacteraceae: Campylobacter jejuni, Campylobacter coli. Global Water Pathogen Project*. Available at: http://www.waterpathogens.org/book/campylobacter 10.3201/eid2212.160841 27869597 PMC5189157

[B60] Polo D. Schaeffer J. Fournet N. Le Saux J.-C. Parnaudeau S. McLeod C. (2016). Digital PCR for quantifying norovirus in oysters implicated in outbreaks, France. *Emerg. Infect. Dis.* 22 2189–2191. 10.3201/eid2212.160841 27869597 PMC5189157

[B61] Potasman I. Paz A. Odeh M. (2002). Infectious outbreaks associated with bivalve shellfish consumption: a worldwide perspective. *Clin. Infect. Dis.* 35 921–928. 10.1086/342330 12355378

[B62] Powell A. Baker-Austin C. Wagley S. Bayley A. Hartnell R. (2013). Isolation of pandemic *Vibrio parahaemolyticus* from UK water and shellfish produce. *Microb. Ecol.* 65 924–927. 10.1007/s00248-013-0201-8 23455432

[B63] Ribot E. M. Fair M. A. Gautom R. Cameron D. N. Hunter S. B. Swaminathan B. (2006). Standardization of pulsed-field gel electrophoresis protocols for the subtyping of *Escherichia coli* O157:H7, *Salmonella*, and *Shigella* for PulseNet. *Foodborne Pathog. Dis.* 3 59–67. 10.1089/fpd.2006.3.59 16602980

[B64] Rodriguez S. Araujo R. (2012). Effect of environmental parameters on the inactivation of the waterborne pathogen Campylobacter in a Mediterranean river. *J. Water Health* 10 100–107. 10.2166/wh.2011.044 22361705

[B65] Rodriguez-Castro A. Ansede-Bermejo J. Blanco-Abad V. Varela-Pet J. Garcia-Martin O. Martinez-Urtaza J. (2010). Prevalence and genetic diversity of pathogenic populations of Vibrio parahaemolyticus in coastal waters of Galicia. *Spain Environ. Microbiol. Rep.* 2 58–66. 10.1111/j.1758-2229.2009.00064.x 23765999

[B66] Ryu H. Grond K. Verheijen B. Elk M. Buehler D. M. Santo Domingo J. W. (2014). Intestinal microbiota and species diversity of *Campylobacter* and *Helicobacter* spp. in migrating shorebirds in Delaware Bay. *Appl. Environ. Microbiol.* 80 1838–1847. 10.1128/AEM.03793-13 24413599 PMC3957654

[B67] Schaeffer J. Le Saux J.-C. Lora M. Atmar R. L. Le Guyader F. S. (2013). Norovirus contamination on French marketed oysters. *Int. J. Food Microbiol.* 166 244–248. 10.1016/j.ijfoodmicro.2013.07.022 23973835 PMC3955953

[B68] Schaeffer J. Treguier C. Piquet J.-C. Gachelin S. Cochennec-Laureau N. Le Saux J.-C. (2018). Improving the efficacy of sewage treatment decreases norovirus contamination in oysters. *Int. J. Food Microbiol.* 286 1–5. 10.1016/j.ijfoodmicro.2018.07.016 30029040

[B69] Semenza J. C. Trinanes J. Lohr W. Sudre B. Löfdahl M. Martinez-Urtaza J. (2017). Environmental suitability of Vibrio Infections in a warming climate: an Early Warning System. *Environ. Health Perspect.* 125:107004. 10.1289/EHP2198 29017986 PMC5933323

[B70] Setti I. Rodriguez-Castro A. Pata M. P. Cadarso-Suarez C. Yacoubi B. Bensmael L. (2009). Characteristics and dynamics of *Salmonella* contamination along the coast of Agadir, Morocco. *Appl. Environ. Microbiol.* 75 7700–7709. 10.1128/AEM.01852-09 19820155 PMC2794122

[B71] Shankar V. Baghdayan A. S. Huycke M. M. Lindahl G. Gilmore M. S. (1999). Infection-derived *Enterococcus faecalis* strains are enriched in esp, a gene encoding a novel surface protein. *Infect. Immun.* 67 193–200. 10.1021/es4050083 9864215 PMC96296

[B72] Sidhu J. Skelly E. Hodgers L. Ahmed W. Li Y. Toze S. (2014). Prevalence of enterococcus species and their virulence genes in fresh water prior to and after storm events. *Environ. Sci. Technol.* 48 2979–2988. 10.1021/es4050083 24494806

[B73] Soares R. O. Fedi A. C. Reiter K. C. Caierão J. d’Azevedo P. A. (2014). Correlation between biofilm formation and gelE, esp, and agg genes in *Enterococcus* spp. clinical isolates. *Virulence* 5 634–637. 10.4161/viru.28998 24782231 PMC4105314

[B74] Stange C. Sidhu J. P. S. Tiehm A. Toze S. (2016). Antibiotic resistance and virulence genes in coliform water isolates. *Int. J. Hyg. Environ. Health* 219 823–831. 10.1016/j.ijheh.2016.07.015 27497615

[B75] Su Y. A. Sulavik M. C. He P. Makinen K. K. Makinen P. L. Fiedler S. (1991). Nucleotide sequence of the gelatinase gene (gelE) from *Enterococcus faecalis* subsp. liquefaciens. *Infect. Immun.* 59 415–420. 10.1016/j.ijfoodmicro.2014.04.016 1846126 PMC257756

[B76] Suffredini E. Mioni R. Mazzette R. Bordin P. Serratore P. Fois F. (2014). Detection and quantification of *Vibrio parahaemolyticus* in shellfish from Italian production areas. *Int. J. Food Microbiol.* 184 14–20. 10.1016/j.ijfoodmicro.2014.04.016 24810197

[B77] Sullender M. E. Baldridge M. T. (2018). Norovirus interactions with the commensal microbiota. *PLoS Pathog.* 14:e1007183. 10.1371/journal.ppat.1007183 30188944 PMC6126856

[B78] Thebault A. Teunis P. F. M. Le Pendu J. Le Guyader F. S. Denis J.-B. (2013). Infectivity of GI and GII noroviruses established from oyster related outbreaks. *Epidemics* 5 98–110. 10.1016/j.epidem.2012.12.004 23746803

[B79] Van Cauteren D. Le Strat Y. Sommen C. Bruyand M. Tourdjman M. Da Silva N. J. (2017). Estimated annual numbers of foodborne pathogen-associated Illnesses, hospitalizations, and deaths, France, 2008-2013. *Emerg. Infect. Dis.* 23 1486–1492. 10.3201/eid2309.170081 28820137 PMC5572882

[B80] Van Dyke M. I. Morton V. K. McLellan N. L. Huck P. M. (2010). The occurrence of Campylobacter in river water and waterfowl within a watershed in southern Ontario, Canada. *J. Appl. Microbiol.* 109 1053–1066. 10.1111/j.1365-2672.2010.04730.x 20408936

[B81] Veljović K. Popović N. Vidojević A. T. Tolinaèki M. Mihajlović S. Jovèić B. (2015). Environmental waters as a source of antibiotic-resistant *Enterococcus* species in Belgrade, Serbia. *Environ. Monit. Assess.* 187:599. 10.1007/s10661-015-4814-x 26314345

[B82] Vereen E. Lowrance R. R. Jenkins M. B. Adams P. Rajeev S. Lipp E. K. (2013). Landscape and seasonal factors influence *Salmonella* and *Campylobacter* prevalence in a rural mixed use watershed. *Water Res.* 47 6075–6085. 10.1016/j.watres.2013.07.028 23969398

[B83] Vignaroli C. Di Sante L. Leoni F. Chierichetti S. Ottaviani D. Citterio B. (2016). Multidrug-resistant and epidemic clones of *Escherichia coli* from natural beds of Venus clam. *Food Microbiol.* 59 1–6. 10.1016/j.fm.2016.05.003 27375238

[B84] Walters S. P. González-Escalona N. Son I. Melka D. C. Sassoubre L. M. Boehm A. B. (2013). *Salmonella enterica* diversity in central Californian coastal waterways. *Appl. Environ. Microbiol.* 79 4199–4209. 10.1128/AEM.00930-13 23624479 PMC3697486

[B85] Wilkes G. Edge T. A. Gannon V. P. J. Jokinen C. Lyautey E. Neumann N. F. (2011). Associations among pathogenic bacteria, parasites, and environmental and land use factors in multiple mixed-use watersheds. *Water Res.* 45 5807–5825. 10.1016/j.watres.2011.06.021 21889781

[B86] Wilson I. G. Moore J. E. (1996). Presence of *Salmonella* spp. and *Campylobacter* spp. in shellfish. *Epidemiol. Infect.* 116 147–153. 8620905 10.1017/s0950268800052377 PMC2271625

[B87] Yoder J. S. Hlavsa M. C. Craun G. F. Hill V. Roberts V. Yu P. A. (2008). Surveillance for waterborne disease and outbreaks associated with recreational water use and other aquatic facility-associated health events–United States, 2005-2006. *MMWR Surveill. Summ.* 57 1–29. 10.1128/AEM.01729-15 18784642

[B88] Yu Y. Cai H. Hu L. Lei R. Pan Y. Yan S. (2015). Molecular epidemiology of oyster-related human noroviruses and their global genetic diversity and temporal-geographical distribution from 1983 to 2014. *Appl. Environ. Microbiol.* 81 7615–7624. 10.1128/AEM.01729-15 26319869 PMC4592855

