# Supplementary material for: Occurrence of Bacterial Pathogens and Human Noroviruses in Shellfish-Harvesting Areas and Their Catchments in France
Source: Front Microbiol. 2018 Oct 11;9:2443. doi: 10.3389/fmicb.2018.02443 (PMC6193098; doi:10.3389/fmicb.2018.02443)
Supplement: Supplementary file 1 [file Data_Sheet_1.pdf]

Supplementary data S1

Supplementary Table 1. Human and livestock density on the catchment areas

Sub-catchment	Human (inhab./km^2)	Pig (pigs/km^2)	Cattle (cattle/km^2)	Poultry (poultry/km^2)	Sheep (sheep/km^2)
Frémur (1A)[a]	ND[b]	1175	56	31129	ND[c]
Le Rat (1B)[a]	ND	414	135	7018	ND[c]
Le Clos (1C)[a]	ND	716	69	4306	ND[c]
Kermiton (1D)[a]	ND	319	14	0	ND[c]
**Mean site 1[a]**	**62**	**967**	**68**	**22780**	**ND[c]**
La Sienne (2A)[d]	ND	56	125	1063	5
La Soules (2B)[d]	ND	161	89	172	3
**Mean site 2[d]**	**39**	**73**	**119**	**921**	**5**
La Vanlée (3A)	ND	19	66	13	25
Les Hardes (3B)	ND	0	126	278	33
**Mean site 3**	**167**	**13**	**84**	**92**	**27**

[a] Data from Jardé et al., 2018.

[b] ND: Not determined.

[c] There is no sheep farm on site 1.

[d] The data are those of an area limited to 30 kms from the seaside (beyond this limit, it is considered that potential sources of pollution have no major impact on the coastal areas of use).

References

Jardé, E., Jeanneau, L., Harrault, L., Quenot, E., Solecki, O., Petitjean, P., et al. (2018). Application of a microbial source tracking based on bacterial and chemical markers in headwater and coastal catchments. *Sci. Total Environ.* 610–611, 55–63. doi:10.1016/j.scitotenv.2017.07.235.
