# Supplementary material for: Occurrence of Bacterial Pathogens and Human Noroviruses in Shellfish-Harvesting Areas and Their Catchments in France
Source: Front Microbiol. 2018 Oct 11;9:2443. doi: 10.3389/fmicb.2018.02443 (PMC6193098; doi:10.3389/fmicb.2018.02443)
Supplement: Supplementary file 2 [file Data_Sheet_2.pdf]

Supplementary data S2.

Detection and characterization of *E. coli* and enterococci

E. coli and enterococci were isolated without an enrichment step to evaluate their diversity. For this purpose, 1, 10, and 100 ml of water were filtered through 0.45 µm cellulose membrane and the filters were put onto TBX agar (Oxoid, Dardilly, France) for *E. coli* and bile esculin azide agar (AES laboratoire) for enterococci. For shellfish, 10 g of homogenized blended SF were distributed onto five empty plates with overlay super-cooled TBX or Slanetz and Bartley (Conda laboratorios) agar. Plates were incubated 24 hours at 44°C for TBX or at 37°C for bile esculin azide agar and Slanetz and Bartley. Presumptive *E. coli* and enterococci were confirmed using mass spectrometry (MALDI-TOF Microflex Bruker, Billerica, USA).

Detection, isolation and characterization of *Salmonella*

Salmonella spp. were investigated using ISO-6579-1:2017 method and detection of *invA* and *ttrBCA* genes in selective enrichments by qPCR (Gonzáles-Escalona et al., 2012). Briefly, 25 g of homogenized blended SF, 10 g of sediment or membranes corresponding to the filtration of 1 liter of water were placed in buffered peptone water (BPW). The suspensions were incubated at 37°C for 18 hours. One hundred microliters of the BPW pre-enrichment were then transferred into 10 ml of Rappaport-Vasilliadis Soya peptone Broth (RVS) for selective enrichment at 41.5°C for 24 hours. In addition, 1 ml of the BPW pre-enrichment was transferred into 10 ml of Muller-Kauffman broth with tetrathionate-novobiocin (MKTTn) for selective enrichment at 37°C for 24 hours. Then, streaking onto selective agar plates: XLD, xylose desoxycholate agar (VWR) and Brilliance *Salmonella* (Oxoid) and incubation at 37°C of the plates for 24 hours were performed. Presumptive colonies (up to five by selective liquid media RVS and MKTTn) were streaked onto nutritive agar plates and incubated at 37°C for 24 hours.

Nucleic acids of these strains were released by heating at 100°C for 10 min and isolates were confirmed as *Salmonella* by detecting *invA* and *ttrCBA* genes specific of the *Salmonella* genus by qPCR (Gonzáles-Escalona et al., 2012).

Detection, isolation and characterization of *Campylobacter*

Campylobacter spp. were investigated using ISO-10272:2016 method and qPCR detection of 16S rRNA genes from enrichment broth (Leblanc-Maridor et al., 2011). Briefly, 25 g of homogenized blended SF, 10 g of sediment or membranes corresponding to the filtration of 1 liter of water were placed in Bolton broth under microaerobic conditions (CampyGen; Oxoid). Then, streaking onto selective agar plates: Casa medium (BioMérieux, Lyon, France) and mCCDA (Oxoid) and incubation at 41.5°C of the plates for 48-72 hours were performed. Up to eight presumptive colonies from these plates were streaked onto Karmali plates (Oxoid) and incubated at 41.5°C for 48-72 hours under microaerobic conditions.

In addition, *Campylobacter* were isolated from water samples using a passive migration method. In this purpose, 50 ml of water were filtered on a 0.2 µm nitrocellulose membrane. The filter was then placed retentate-down on a 0.45 µm cellulose membrane previously deposited on Casa agar. After incubation under microaerobic conditions at 41.5°C for 18 hours, filters were removed from the agar surface and plates were reincubated another 48 h.

Detection, isolation and characterization of *Vibrio*

Presence of *Vibrio parahaemolyticus*, *V. cholerae,* and *V. vulnificus* in a selection of samples including shellfish and seawater was evaluated using ISO-21872:2017 method and detection of *toxR* gene, 16S-23S intergenic spacer (IGS) region and *vvhA* gene in selective enrichments by qPCR or PCR (Hervio-Heath et al., 2012, Chun et al., 1999, Lee et al., 1997). Primers specific for *tdh* and *trh* genes were used for the identification of haemolysin-producing and potentially pathogenic *V. parahaemolyticus* (Hervio-Heath et al., 2002). Briefly, 25 g of homogenized blended SF or membranes corresponding to the filtration of 1 liter of seawater were placed in alkaline peptone water 2% NaCl (APW, pH 8.6) and incubated at 41.5°C for 6 hours. One ml of the APW pre-enrichment was transferred in 10 ml APW and incubated at 41.5°C for 18 hours. The APW-enriched cultures were used for DNA extraction and *V. parahaemolyticus*, *V. cholerae* and *V. vulnificus* isolations.

References

Chun, J., Huq, A., and Colwell, R. R. (1999). Analysis of 16S-23S rRNA intergenic spacer regions of *Vibrio cholerae* and *Vibrio mimicus*. *Appl. Environ. Microbiol.* 65, 2202–2208.

González-Escalona, N., Brown, E. W., and Zhang, G. (2012). Development and evaluation of a multiplex real-time PCR (qPCR) assay targeting *ttrRSBCA* locus and *invA* gene for accurate detection of *Salmonella* spp. in fresh produce and eggs. *Food Research International* 48, 202–208. doi:10.1016/j.foodres.2012.03.009.

Hervio-Heath, D., Colwell, R. R., Derrien, A., Robert-Pillot, A., Fournier, J. M., and Pommepuy, M. (2002). Occurrence of pathogenic vibrios in coastal areas of France. *J. Appl. Microbiol.* 92, 1123–1135.

Hervio-Heath, D., Constantin de Magny, G., Deter, J., Teillon, A., Lozach, S., and Derrien, A. (2012). A survey of *Vibrio parahaemolyticus* in French Atlantic coastal waters. *In Pathogenic Vibrio spp. in Northern European Waters: International Symposium.* 11–16.

ISO-10272:2016. Microbiology of food and animal feeding stuffs: Horizontal method for detection and enumeration of *Campylobacter* spp. -Part 1: Detection method (https://www.iso.org/standard/37091.html). -Part 2: Colony-count technique (https://www.iso.org/standard/63228.html).

ISO-21872:2017. Microbiologie de la chaîne alimentaire: Méthode horizontale pour la détermination des *Vibrio* spp. -Partie 1: Recherche des espèces de *Vibrio parahaemolyticus, Vibrio cholerae* et *Vibrio vulnificus* potentiellement entéropathogènes (https://www.iso.org/fr/standard/74112.html). -Partie 2: Recherche des espèces autres que *Vibrio parahaemolyticus* et *Vibrio cholerae* (https://www.iso.org/fr/standard/38279.html).

ISO-6579-1:2017. Microbiology of the food chain - Horizontal method for the detection, enumeration and serotyping of *Salmonella* -Part 1: Detection of *Salmonella* spp (https://www.iso.org/fr/standard/56712.html).

Leblanc-Maridor, M., Beaudeau, F., Seegers, H., Denis, M., and Belloc, C. (2011). Rapid identification and quantification of *Campylobacter coli* and *Campylobacter jejuni* by real-time PCR in pure cultures and in complex samples. *BMC Microbiol.* 11, 113.

doi:10.1186/1471-2180-11-113.

Lee, J. Y., Eun, J. B., and Choi, S. H. (1997). Improving Detection of *Vibrio vulnificus* in *Octopus variabilis* by PCR. *Journal of Food Science* 62, 179–182. doi:10.1111/j.1365-2621.1997.tb04395.x.
