# Supplementary material for: Occurrence of Bacterial Pathogens and Human Noroviruses in Shellfish-Harvesting Areas and Their Catchments in France
Source: Front Microbiol. 2018 Oct 11;9:2443. doi: 10.3389/fmicb.2018.02443 (PMC6193098; doi:10.3389/fmicb.2018.02443)
Supplement: Supplementary file 3 [file Data_Sheet_3.pdf]

Supplementary data S3

Supplementary Table 2. Primers used in this study

Primer	Sequence (5'-3')	Reference	Size of amplimer (bp)
**Primers used for classification of *E. coli* in phylogenetic groups**			
chuA.1	GACGAACCAACGGTCAGGAT	Clermont et al., 2000	279
chuA.2	TGCCGCCAGTACCAAAGACA	Clermont et al., 2000	
yjaA.2	ATGGAGAATGCGTTCTCAAC	Clermont et al., 2000	168
yjaabisF	ATCGCCAATTTCTTTGTTGC	This study	
tspE4C2.1	GAGTAATGTCGGGGCATTCA	Clermont et al., 2000	152
tspE4C2.2	CGCGCCAACAAAGTATTACG	Clermont et al., 2000	
**Primers used for detection of virulence genes in enterococci**			
RTespL	GAGTTAGCGGGAACAGGTCA	This study	101
RTespR	ATTGGAGCCCCATCTTTTTC	This study	
gelED	AGTTCATGTCTATTTTCTTCAC	This study	203
RTgelE	ACAGTAACGCCTTCCGTTTG	This study	
RTaggL	TGATGAAAACCAACGCAGAC	This study	71
aggR	AAACGGCAAGACAAGTAAATA	This study	

References

Clermont, O., Bonacorsi, S., and Bingen, E. (2000). Rapid and simple determination of the *Escherichia coli* phylogenetic group. *Appl. Environ. Microbiol.* 66, 4555–4558.
