# Supplementary material for: Occurrence of Bacterial Pathogens and Human Noroviruses in Shellfish-Harvesting Areas and Their Catchments in France
Source: Front Microbiol. 2018 Oct 11;9:2443. doi: 10.3389/fmicb.2018.02443 (PMC6193098; doi:10.3389/fmicb.2018.02443)
Supplement: Supplementary file 4 [file Data_Sheet_4.pdf]

Supplementary data S4.

Characterization of *E. coli*

7,452 *E. coli* strains were isolated; 3,043 from site 1, 2,635 from site 2, and 1,774 from site 3. Among them, 4,579 isolates came from river water, 758 from seawater, and 2,115 from shellfish. Among the 2,089 *E. coli* isolates from phylogroups B2 and D, 2,024 strains (1,146 from D and 876 from B2) were analyzed for the presence of nine virulence genes (*fyuA, hlyC, sfa, papC, iucC, papGIII, cnf1, papGII,* and *iroN*). Site-by-site analysis revealed a higher percentage of strains from site 1 carrying the *fyuA*, *sfa*, or *papGIII* genes compared to strains from the other sites (Supplementary Figure 1A). The mean virulence scores (number of virulence factors present among the nine tested) was 3.7 for isolates from site 1 whereas it was 3.0 and 2.9 respectively for strains from sites 2 and 3. This difference was particularly marked for isolates from shellfish since the virulence scores were 3.7, 2.8 and 2.5, respectively for sites 1, 2 and 3. Strains with four or more virulence genes represent 55.1% of the *E. coli* isolated from shellfish in site 1 against 38.0% for the isolates from shellfish collected in the other sites. The mean virulence score was higher for strains belonging to B2 phylogroup (3.9) than for those of phylogroup D (3.0). Except for *hlyC* and *iucC* genes which harbored at a similar percentage by the strains of phylogroups B2 and D, for all the other genes the percentage of strains harboring each virulence factor was significantly higher among phylogroup B2 isolates (data not shown).

Four hundred and fourty nine *E. coli* strains were analyzed to evaluate their level of resistance against a panel of 16 antibiotics or combinations of antibiotics. Of all tested isolates, the percentage of clones resistant to a given antibiotic varies from 0.4% for imipenem and amikacin to 12.2% for amoxicillin (Supplementary Figure 1B). Antibiotics for which a higher rate of resistant strains were observed are amoxicillin (12.2%), doxycycline (11.8%) and ticarcillin (7.8%). In order to identify a potential source of antibiotic-resistant bacteria, we focused on antibiotics for which the percentage of resistant bacteria was higher than 10 for at least one of the water samples. These are cefalotin, cefoxitin, doxycycline, amoxicillin plus clavulanic acid, ticarcillin and amoxicillin. Compared to isolates from other rivers, the results showed that a higher proportion of *E. coli* from Les Hardes river were resistant to cefoxitin (19.4% *versus* 6.3%), amoxicillin + clavulanic acid (19.4% *vs* 5.0%), and amoxicillin (27.8% *vs* 12.2%). On the other hand, isolates from Le Frémur river were statistically more frequently resistant to doxycycline (22.2% *vs* 11.3% for other rivers). The highest rates of antibiotic resistance among the 146 strains from shellfish were for doxycycline (11.0%), amoxicillin (9.6%) and ticarcillin (5.5%).

Characterization of enterococci

Two thousand sixty-nine enterococcal strains (1,200 *E. faecalis* and 869 *E. faecium*) were analyzed for the presence of three virulence genes (*esp, gelE*, and *agg*). These genes were more frequently detected within the species *E. faecalis* than *E. faecium*. Indeed, genes *esp, gelE,* and *agg* occurred in 34.9%, 49.8% and 46.0% of *E. faecalis* strains, respectively while they were detected in 4.8%, 3.2% and 2.2% of *E. faecium* isolates, respectively (Supplementary Figures 2A and 2B). Their occurrence was comparable from one site to another, except for *gelE*, which was more frequently detected in *E. faecalis* from site 1 (56.3%) than from other sites (45.8%). The percentage of bacteria carrying *gelE* was lower among *E. faecalis* isolated from shellfish (44.3%) compared to those from water samples (54.3%). It was the same for *E. faecium* strains carrying *esp* (1.8% *versus* 6.7%) and *gelE* (1.5% *versus* 4.3%).

Susceptibility of 213 *E. faecalis* and 233 *E. faecium* strains to 16 antibiotics was determined. Considering both species, the most frequently observed resistance was against lincomycin (51.1%) followed by doxycycline (21.1%), erythromycin (20.4%) and levofloxacin (19.7%). The percentages of strains resistant to these antibiotics were 48.8%, 22.7%, 20.8%, and 23.8%, respectively for enterococci from water samples and 54.0%, 19.1%, 19.1% and 13.7%, respectively for isolates from shellfish. *E. faecalis* resistance, ranged from 0 % for ampicillin to 74.2 % for lincomycin whereas for *E. faecium* it ranged from 0.4 % for vancomycin and teicoplanin to 32.1 % for levofloxacin (Supplementary Figure 2C). Interestingly, the percentage of *E. faecium* resistant to levofloxacin, ampicillin and streptomycin was higher for strains isolated from water samples (37.8%, 29.4% and 13.3 %, respectively) than from shellfish (22.0%, 4.6% and 3.4 %, respectively). In order to provide information about the origin of antibiotic-resistant strains, we focused on the susceptibility of strains from river samples. Interestingly, 64.3 % of *E. faecium* strains from La Sienne river (42 isolates tested) and 55.5% from La Soules (27 isolates tested) were resistant to ampicillin, whereas all 47 strains of this species that have been isolated from the other rivers were sensitive to this antibiotic (data not shown). On the other hand, of the 42 *E. faecium* strains isolated from La Sienne and tested for antibiotic susceptibility, 80.9% were resistant to levofloxacin, while for other rivers the corresponding percentage was less than or equal to 40.0% (data not shown).

Figures

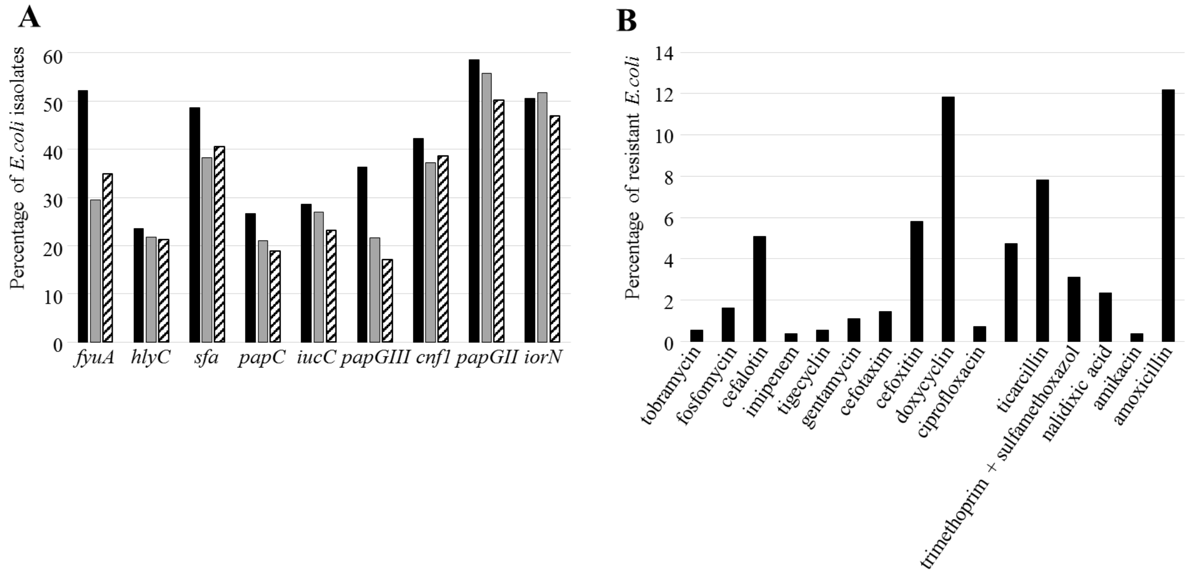

Supplementary Figure 1. Observed prevalence of virulence genes (A) and antimicrobial susceptibility testing (B) of *E. coli*. Prevalence of virulence genes is represented by black, gray and hatched bars for sites 1, 2 and 3, respectively.

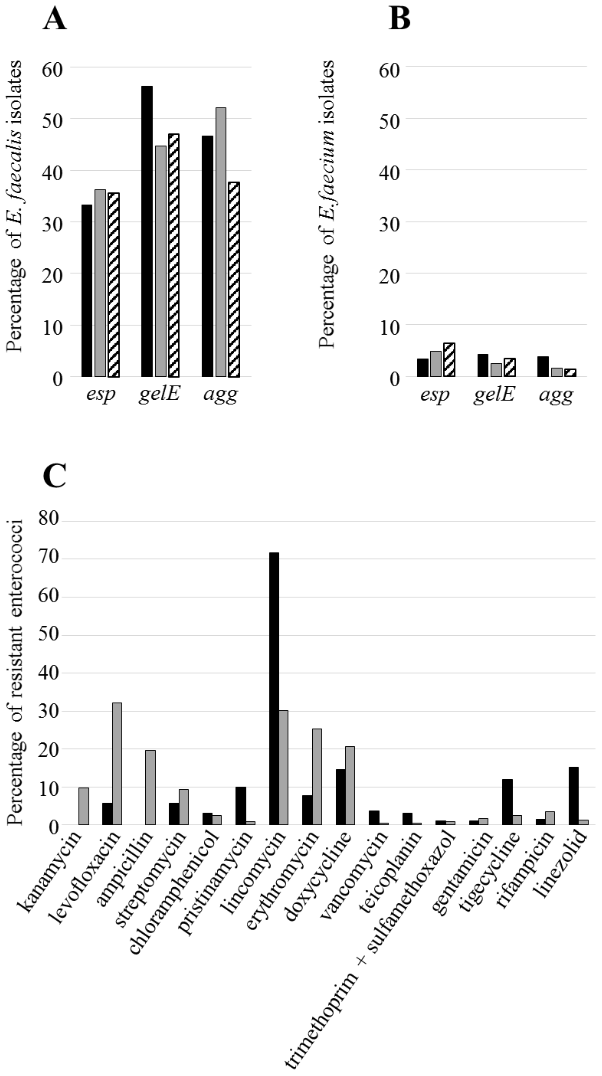

Supplementary Figure 2. Observed prevalence of virulence genes (A and B) and antimicrobial susceptibility testing (C) of enterococci. On panels A and B, black, gray and hatched bars represent results from sites 1, 2 and 3, respectively. On panel C, *E. faecalis* and *E. faecium* are represented by black and gray bars, respectively.
